# Bioactive Compounds, Antioxidant, Antimicrobial and Anticancerogenic Activity in *Lacmellea edulis* H. Karst., at Different Stages of Maturity

**DOI:** 10.3390/antiox14101232

**Published:** 2025-10-14

**Authors:** Elena Coyago-Cruz, Johana Zúñiga-Miranda, Gabriela Méndez, Melany Alomoto, Steven Vélez-Vite, Carlos Barba-Ostria, Rebeca Gonzalez-Pastor, Jorge Heredia-Moya

**Affiliations:** 1 Carrera de Ingeniería en Biotecnología, Universidad Politécnica Salesiana, Sede Quito, Campus El Girón, Av. 12 de Octubre N2422 y Wilson, Quito 170143, Ecuadorsvelezv2@est.ups.edu.ec (S.V.-V.); 2 Centro de Investigación Biomédica (CENBIO), Facultad de Ciencias de la Salud Eugenio Espejo, Universidad UTE, Quito 170527, Ecuador; 3 Escuela de Medicina, Colegio de Ciencias de la Salud Quito, Universidad San Francisco de Quito (USFQ), Quito 170901, Ecuador; 4 Instituto de Microbiología, Universidad San Francisco de Quito (USFQ), Quito 170901, Ecuador

**Keywords:** chicle fruit, functional food, antibacterial activity, antifungal activity, cell cultures, tumour cell lines

## Abstract

*Lacmellea edulis*, traditionally known as chicle, is a species that has received little attention despite its nutraceutical potential. This study aimed to evaluate the physicochemical characteristics (pH, soluble solids, titratable acidity, moisture, ash, and minerals) and the content of bioactive compounds (vitamin C, organic acids, carotenoids, and phenols) of fruits at different stages of ripeness, as well as their antimicrobial (against *Candida albicans*, *Candida tropicalis*, *Escherichia coli*, *Staphylococcus aureus*, and *Streptococcus mutans*), antiproliferative and antihaemolytic activity. Bioactive compounds were quantified using liquid chromatography, while biological activities were assessed via spectrophotometric assays. The results revealed a high concentration of ascorbic acid in the ripe pulp (3.0 mg/100 g DW), higher levels of organic acids in the unripe pulp (3947.6 mg/100 g DW), and a high total phenol content in the ripe peel (10,890.9 mg/100 g DW). The peel exhibited the highest antioxidant activity (63.3 mmol ET/100 g DW). Regarding antimicrobial activity, the pulp exhibited the lowest MIC values against *E. coli* (2.7 mg/mL) and *S. mutans* (2.6 mg/mL), the peel against *S. aureus* (21.3 mg/mL) and *C. tropicalis* (5.3 mg/mL), and the seeds against *C. albicans* (20.8 mg/mL). Additionally, the peel exhibited the greatest antiproliferative efficacy against cervical (HeLa) and hepatoma (HepG2) cancer cells. None of the evaluated extracts showed significant haemolytic effects, confirming their safety. Overall, *L. edulis* appears to be a promising source of bioactive metabolites with potential applications in functional foods and pharmaceutical products.

## 1. Introduction

Biodiversity is a fundamental pillar of ecosystem stability and human well-being. It encompasses the wide variety of life forms present on Earth [1]. Much of this biodiversity is concentrated in tropical regions, which are considered reservoirs of biological diversity. However, the loss of biodiversity is an increasing threat that undermines food security, increases vulnerability to natural disasters, and reduces the quality of life of populations. In this context, nutrition, health and environmental sustainability are closely interlinked within food systems [2].

At the same time, there have been significant shifts in global dietary patterns over recent decades. The consumption of ultra-processed foods has increased considerably worldwide [3]. This trend intensified during the pandemic, with reports of increased food intake, weight gain, and altered eating habits during lockdowns [4]. In response, regional diets such as the Mediterranean and New Nordic diets have been shown to offer significant health benefits. Emerging research suggests that nutrition plays a pivotal role in mental health and that factors such as balanced diets, physical activity, and antioxidant intake possess significant therapeutic potential [5].

In addition to nutritional challenges, pathogenic microorganisms have developed resistance to commonly used antimicrobials, posing a critical threat to global public health [6,7]. The World Health Organisation has identified the ESKAPE pathogens (*Enterococcus faecium*, *Staphylococcus aureus*, *Klebsiella pneumoniae*, *Acinetobacter baumannii*, *Pseudomonas aeruginosa* and *Enterobacter* species) as a high priority due to their resistance mechanisms [6]. This resistance is driven by factors including the excessive and inappropriate use of antibiotics in human health and agriculture, as well as socioeconomic and environmental changes [7,8].

Against this backdrop, natural compounds have regained relevance as an alternative source of drugs. Although their study declined at the end of the 20th century due to the emergence of new synthetic strategies, they are now re-emerging as valuable contributors to health research [7,9]. In this regard, consuming fruits and their extracts is a promising strategy for mitigating antimicrobial resistance and promoting overall health. While some fruits may harbour antibiotic-resistant bacteria, their phytochemicals exhibit antimicrobial properties against various pathogens [10,11]. Antioxidant compounds contribute to this effect by altering bacterial cell membranes and suppressing essential metabolic activities [11].

Fruit by-products, such as peels and seeds, are also a rich source of bioactive compounds that offer a variety of benefits. For instance, mangoes have been reported to contain polyphenols and carotenoids with antioxidant and antimicrobial properties [12], while tree tomato peels have been found to have high concentrations of phenolic compounds [13]. Similarly, the seeds of tropical fruits such as mango, durian, and jackfruit contain polyphenols, flavonoids, and carotenoids of significant functional value [14]. The development and ripening process of fruits modulates the synthesis of phytochemicals, such as phenols, terpenoids, and nitrogen- or sulfur-containing compounds. These not only contribute to sensory attributes, but also participate in disease prevention [15]. These bioactive molecules have demonstrated antioxidant, anti-inflammatory, and anti-ageing properties, which are linked to the prevention of chronic diseases such as cancer and diabetes [16]. Mesoamerican fruits in particular have demonstrated a wide range of bioactivities [17]. Furthermore, the valorisation of fruit waste provides opportunities to obtain ingredients for use in nutraceuticals and functional foods [18,19].

In this context, the Apocynaceae family is significant as it comprises around 250 genera and over 2000 species distributed across five subfamilies, including Apocynoideae, Asclepiadoideae, Periplocoideae, Rauvolfoideae, and *Secamonoideae*. This family constitutes a significant reservoir of secondary metabolites. The family’s species range from shrubs to trees and have traditionally been used to treat fever, malaria, gastrointestinal disorders and diabetes [9]. The medicinal importance of this family lies in its alkaloid content, which has applications in cancer therapies. However, several of its fruits remain largely unexplored. One such example is *Lacmellea standleyri*, also known as ‘Lechemiel’, which is consumed by local indigenous communities in South America but has received little commercial attention [20], similar to the case in *Lacmellea oblonga* [21].

In this context, *Lacmellea edulis*, a species belonging to the subfamily Rauvolfioideae, is of interest. Its fruits are 2–2.2 cm in size, broad and ellipsoid, and turn a distinctive yellow colour when ripe. It grows in humid forests in Mesoamerica and South America, including Colombia, Ecuador, Venezuela, Peru and Brazil [22]. Despite its presence in these regions, the fruit is poorly studied and virtually unknown in biochemical and pharmacological research. The objective of this study was therefore to characterise the bioactive compound profile in *L. edulis* at different stages of ripeness, and to analyse its correlation with biological activities of interest, such as antimicrobial, antihaemolytic, and anticancer activity. This will provide new knowledge about the nutraceutical and pharmacological value of this species. The findings of this study aim to contribute to the revaluation of little-explored native species and strengthen the basis for their potential industrial use.

## 2. Materials and Methods

### 2.1. Reagents and Standards

Ultra-pure water used for all procedures was generated using a NANOpure Diamond™ system (Barnstead Inc., Dubuque, IA, USA). Microbial culture media were selected according to standard microbiological protocols; thus, Sabouraud Dextrose Broth (YPDB), Brain Heart Infusion (BHI) and Muller Hinton Agar (MHA) were supplied by BD Difco™ (Fisher Scientific Inc., Madrid, Spain). Yeast peptone dextrose Broth (YPDB) was purchased from Sisco (Sisco Research Laboratories, Bombay, India). And streptomycin sulfate (CAS 3810-74-0) was obtained from Phytotech (PhytoTechnology Laboratories ^®^, Lenexa, KS, USA). The yeast microbial strains employed in this study included *Candida albicans* ATCC 1031 and *Candida tropicalis* ATCC 13803. Bacterial strains analysed for antimicrobial activity included *Escherichia coli* ATCC 8739, *Pseudomonas aeruginosa* ATCC 9027, *Staphylococcus aureus* ATCC 6538P, and *Streptococcus mutans* ATCC 25175. All strains were purchased from the American Type Culture Collection (ATCC, Manassas, VA, USA). Sheep blood was obtained from a certified local supplier in Quito, Ecuador, that follows stringent protocols for safe blood collection and processing, ensuring compliance with biosafety and ethical guidelines.

Standards and reference compounds were employed to support chemical profiling. These included a broad range of phenolic acids, flavonoids, carotenoids, and antioxidants, all of which were of certified purity and acquired from Sigma (Merck, Darmstadt, Germany). Notably compounds included β-cryptoxanthin (CAS 472-70-8, 97.0%), citric acid (CAS 77-92-9, 100.8%), *L*-(+)-ascorbic acid (CAS 50-81-7, 99.8%), gallic acid (CAS 149-91-7, 100%), ferulic acid (CAS 1135-24-6, 100%), caffeic acid (CAS 331-39-5,, 98.0%), shikimic acid (CAS 138-59-0, 99.0%), malic acid (CAS 97-67-6, 99.0%) and *L*-(+)-tartaric acid (CAS 87-69-4, 99.5%), kaempferol 97.0% (CAS 520-18-3), naringin (CAS 10236-47-7, 95.0%), rutina (CAS 153-18-4, 94.0%) luteolin (CAS 491-70-3, 98.0%) and quercetin (CAS 84906-19-8, 95.0%). Other compounds of interest were chrysin (CAS 480-40-0, 97.%), chlorogenic acid (CAS 327-97-9, 95.0%), *p*-coumaric acids (CAS 501-98-4), *m*-coumaric acids (CAS 588-30-7), *o*-coumaric acids (CAS 614-60-8), 3-hydroxybenzoic acid (CAS 99-06-3, 99%), 2,5-dihydroxybenzoic acid (CAS 490-79-9, 98%), *p*-hydroxybenzoic acid (CAS 99-06-3, 99%), syringic acid (CAS 530-57-4 95%), vanillic acid (CAS 121-34-6, 97.0%), β-carotene 93.0% (CAS 7235-40-7), lutein (CAS 127-40-2), lycopene (CAS 502-65-8), zeaxanthin (CAS 144-68-3), β-cryptoxanthin (CAS 472-70-8, 97.0%), chlorophyll a (CAS 479-61-8), chlorophyll b (CAS 519-62-0) and Trolox (CAS 53188-07-1, 98.0%).

Metal standards used for mineral analysis were iron (CAS 7439-89-6), magnesium (CAS7439-95-4), potassium (CAS7440-09-7), calcium (CAS7440-70-2), and sodium (CAS7440-23-5) with a concentration of 1000 μg/mL, provided from Accustandard (AccuStandard, Inc., New Haven, CT, USA).

Chemical reagents for antioxidant assays and sample preparation were of analytical grade included *DL*-homocysteine (CAS 454-29-5), DPPH (2,2-diphenyl-1-picrylhydrazyl) (CAS 1898-66-4), ABTS (2,2′-azino-bis-(3-ethylbenzothiazoline-6-sulfonic acid) (CAS 30931-67-0), metaphosphoric acid (CAS 37267-86-0), formic acid (CAS 64-18-6), nitric acid (CAS7697-37-2), potassium chloride (CAS7447-407), *n*-acetyl-*n,n,n*-trimethylammonium bromide (CAS 57-09-0), potassium monobasic phosphate (CAS 7778-77-0), potassium persulfate (CAS 7727-21-1), sulfuric acid (CAS 7664-93-9) and sodium hydroxide (CAS1310-73-2). All were obtained from Sigma-Aldrich.

Finally, organic solvents essential for chromatographic procedures were used in HPLC grade. These included fluconazole (CAS 8638673-4), acetone (CAS 67-64-1), trichloromethane (CAS 67-66-3), ethanol (CAS 64-17-5), acetonitrile (CAS 75-05-8), ethyl acetate (CAS 141-78-6) and methanol (CAS 67-56-1), which were purchased from Fisher Chemical (Fisher Scientific Inc., Madrid, Spain).

### 2.2. Physico-Chemical Analyses

**Figure 1:**
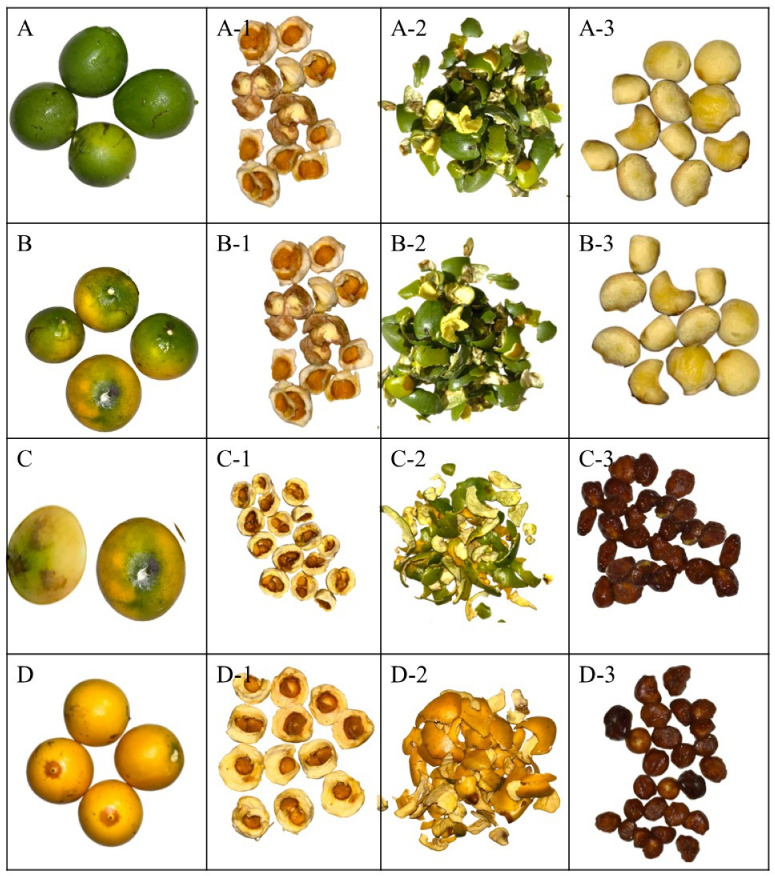
*Lacmellea edulis* H. Karst. (Chicle) at different stages of maturity. Note: (**A**) M0%, physiological maturity with 0% yellow colour in the peel; (**B**) M50%, commercial maturity with 50% yellow colour in the peel; (**C**) M80%, commercial maturity with 80% yellow colour in the peel; (**D**) M100%, ripe fruit with 100% yellow colour in the peel; (**A-1**,**B-1**,**C-1**,**D-1**), Pulp to the respective stages of maturity; (**A-2**,**B-2**,**C-2**,**D-2**), Peel at the respective stages of maturity; (**A-3**,**B-3**,**C-3**,**D-3**), Seeds at their respective stages of maturity.

The study considered chicle fruits (*Lacmellea edulis* H. Karst.) at various stages of maturity (Figure 1). Thus, M0% corresponded to physiological maturity with 0% yellow colour in the peel, M50% to commercial maturity with 50% yellow colour in the peel, M80% to commercial maturity with 80% yellow colour in the peel, and M100% to ripe fruit with 100% yellow colour in the peel. Samples were collected in the province of Pichincha in Ecuador (geographical coordinates: 0°4′52.8″ S, 79°2′56.3″ W). To confirm the taxonomic identity of the species, botanical samples were collected and subsequently authenticated in the herbarium of the Universidad Politécnica Salesiana (Identification code: 04502, Herbario QUPS-Ecuador).

A total of forty fruits for each ripening stage was collected at random from a single production area to ensure representativeness. The sample was divided into two portions. The first portion (twenty fruits) was used to evaluate the physicochemical parameters, which included measurements of fresh weight. To assess chemical properties, the pH was measured using a SevenMulti S47 (Mettler Toledo, Columbus, OH, USA), and soluble solids content was determined with a handheld refractometer (Hitech RHB-32, G-Won Hitech Co., Ltd., Seoul, Republic of Korea), following the standardised ISO 2173 procedure [23]. Titratable acidity was quantified through classical acid–base titration methods. Moisture content was established at 100 °C in a Memmert Be 20 oven (Memmert GmbH + Co. KG, Barcelona, Spain). Ash content was determined through incineration in a muffle furnace at 550 °C (Thermo Fisher Scientific, Waltham, MA, USA) [17].

The second portion (20 fruits) was separated into pulp, peel, and seeds. A composite sample was taken from each fraction, which was then frozen at –80 °C to subsequent freeze-drying using a Christ Alpha 1–4 LDplus (GmbH, Osterode am Harz, Germany). Once dehydrated, the samples were finely ground and stored in amber glass vials under a nitrogen atmosphere to prevent oxidative degradation until further analysis was conducted.

#### Mineral Profile

For mineral extraction, 40 mg of the lyophilised sample was accurately weighed and placed into a microwave digestion vessel, specifically a Speedwave Xpert microwave digestion vessel (Berghof Products + Instruments GmbH, Eningen under Achalm, Germany). The sample was combined with 5 mL of concentrated nitric acid (65%) and allowed to pre-react for 10 min at ambient temperature–pressure program: an initial phase at 140 °C and 30 bar for 5 min at 70% power, followed by 15 min at 200 °C and 35 bar at 80% power, and finally a cooling phase at 50 °C and 25 bar for 10 min with no applied power. Once digestion was complete, the vessels were left to cool at room temperature for 20 min. The resulting solution was transferred into a 25 mL volumetric flask, rinsed with Milli-Q water, brought to volume, and stored in amber glass containers under dark conditions until analysis [24].

Quantification of mineral content was carried out using flame atomic absorption spectrometry (AAS) with a Varian SpectrAA-55 atomic absorption spectrophotometer (Agilent Technologies, Mississauga, ON, Canada). Calibration curves were constructed from 1000 ppm stock standards, with the following working concentration ranges: 0–5 ppm for Ca, 0–20 ppm for Fe, 0–200 ppm for K, 0–10 ppm for Mg, and 0–8 ppm for Na. Final mineral contents were reported as milligrams per 100 g of dry weight (mg/100 g DW) [21].

### 2.3. Analysis of Bioactive Compounds

#### 2.3.1. Ascorbic Acid

To extract the level of *L*-ascorbic acid, 40 mg of freeze-dried fruit powder was combined with 1.2 mL of 3% metaphosphoric acid and 0.2 mL of 0.2% DL-homocysteine solution. The mixture was vortexed thoroughly using a VM-300 mixer (Interbiolab Inc., Orlando, FL, USA) to ensure complete dispersion. The mixture was subjected to ultrasonic extraction in an FS60 ultrasonic bath (Fisher Scientific Inc., Waltham, MA, USA) at a frequency of 40 kHz and a temperature of 25 °C for three minutes. Mili-Q ultrapure water was then added to bring the final volume of the extract to 2 mL. The extraction was performed in triplicate.

The resulting solution was passed through a 0.45 µm PVDF filter and analysed using a Rapid Resolution Liquid Chromatography 1200 RRLC (Agilent Technologies, Mississauga, ON, Canada) equipped with a Diode Array Detector (DAD-UV-VIS), operating at a detection wavelength of 244 nm. Separation was carried out on a Zorbax Eclipse XDB 80 Å C18 Column (1.8 μm, 4.6 mm × 50 mm) (Agilent Scientific Instruments, Santa Clara, CA, USA). The mobile phase, which had a flow rate of 1 mL/min, consisted of a solution containing 90% aqueous phosphate (1.5% monobasic potassium phosphate) and 10% methanolic bromide (1.8% *n,n,n*-trimethyl-*n*-acetylammonium bromide). The chromatograms were retrieved using OpenLab ChemStation software (version 2.15.26). *L*-ascorbic acid was identified by comparing its retention time, UV–visible spectrum, and an internal standard. Quantification was based on a calibration curve constructed from standard ascorbic acid solutions (0.1 mg/mL), injected at varying volumes ranging from 2 to 20 µL. Results were expressed as milligrams of ascorbic acid per 100 g of dry weight (mg/100 g DW). All measurements were performed in triplicate to ensure reproducibility [13].

#### 2.3.2. Organic Acid Profile

Organic acids were extracted from the lyophilised samples by weighing 40 mg of powdered material into a reaction tube and adding 1.5 mL of an extraction solution composed of 0.02 N sulfuric acid, 0.05% metaphosphoric acid and 0.02% *DL*-homocysteine. The mixture was thoroughly homogenised and then subjected to ultrasonic treatment for 3 min in an FS60 ultrasonic bath (Fisher Scientific Inc., Waltham, MA, USA) with a 40 kHz frequency and a temperature of 25 °C to enhance extraction efficiency. The extraction was performed in triplicate.

Following this process, the solution was passed through a 0.45 µm PVDF filter to remove particulates before chromatographic analysis. Quantification was performed using an RRLC system 1200 (Agilent Technologies, Mississauga, ON, Canada) fitted with a Diode Array Detector (DAD-UV-VIS), with detection set at 210 nm. Separation of the organic acids was achieved using a YMC-Triart C18 column (3 µm, 4.6 nm × 150 nm) (YMC Europe GmbH, Dinslaken, Germany). The mobile phase, which had a flow rate of 1 mL/min, consisted of 0.027% sulphuric acid. The chromatograms were retrieved using OpenLab ChemStation software (version 2.15.26). Organic acids were identified by comparing their retention time, UV–visible spectrum, and an internal standard. Calibration curves were established using individual standard solutions (100 mg/mL) of citric, malic, and tartaric acids, which were injected in varying volumes from 2 to 20 µL. Results were reported as milligrams of organic acid per 100 g of dry weight (mg/100 g DW). All determinations were conducted in triplicate to ensure accuracy and reproducibility [13].

#### 2.3.3. Carotenoid Profile

Carotenoids were extracted from lyophilised samples. A 20 mg portion of freeze-dried powder was mixed with a solvent mixture consisting of methanol, acetone, and dichloromethane in a 1:1:2 volume ratio. The sample was homogenised and subjected to ultrasonic agitation for 2 min in an FS60 ultrasonic bath (Fisher Scientific Inc., Waltham, MA, USA) with a 40 kHz frequency and a temperature of 25 °C. The supernatant was recovered by centrifugation at 14,000× *g* rpm for 5 min at 4 °C. This extraction cycle was repeated as needed until all pigmented compounds were visibly removed from the solid matrix. The resulting-coloured phase was collected according to the method described by Coyago-Cruz et al. [17]. The extraction was performed in triplicate.

The dried pigment extract was reconstituted in 40 µL of ethyl acetate and injected into a high-performance liquid chromatography system 1200 RRLC (Agilent Technologies, Mississauga, ON, Canada), equipped with a Diode Array Detector (DAD-UV-VIS) and a YMC C30 column (3 μm, 4.6 × 150 mm; YMC Europe GmbH, Dinslaken, Germany). The mobile phase, which had a flow rate of 1 mL/min, consisted of acetonitrile (solvent A), methanol (solvent B), and ethyl acetate (solvent C) pumped to 85% A + 15% B at 0 min; 60% A + 20% B + 20% C at 5 min; 60% A + 20% B + 20% C at 7 min; 85% A + 15% B at 9 min; 85% A + 15% B at 12 min. The chromatograms were retrieved using OpenLab ChemStation software (version 2.15.26). Carotenoids were identified by comparing their retention times, UV–visible spectra, and internal standards, and monitored at 285, 350, and 450 nm. Individual carotenoids, including astaxanthin, β-carotene, α-carotene, β-cryptoxanthin, lutein, trans-β-apo-8-carotenal, lycopene, zeaxanthin and violaxanthin were identified and quantified by comparison with standards. Total carotenoid content was calculated as the sum of all quantified compounds and expressed in milligrams per 100 g of dry weight (mg/100 g DW).

#### 2.3.4. Chlorophylls and Their Derivatives

Chlorophyll pigments and their degradation products were extracted and quantified following the procedure outlined previously (see Section 2.3.3). For quantification purposes, calibration curves were prepared using standard solutions of chlorophyll a 0.1 mg/mL, pheophytin b and pheophytin a. Injection volumes ranging from 2 to 20 µL were used to generate standard curves.

Concentrations of the pigments in the samples were determined by comparison with standard curves, and the results were expressed as milligrams of compound per 100 g of dry sample (mg/100 g DW).

#### 2.3.5. Phenol Profile

The extraction and quantification of phenolic compounds were conducted according to the method described by Coyago-Cruz et al. [17]. The extraction was performed in triplicate. For each analysis, 20 mg of freeze-dried sample powder was suspended in 100 µL of 80% methanol acidified with 0.1% hydrochloric acid. The sample was homogenised and subjected to ultrasonic agitation for 3 min in an FS60 ultrasonic bath (Fisher Scientific Inc., Waltham, MA, USA) with a 40 kHz frequency and a temperature of 25 °C. The supernatant was recovered by centrifugation at 14,000× *g* rpm for 5 min at 4 °C. The remaining pellet was re-extracted twice more with 500 µL portions of the same solvent. All collected extracts were pooled, filtered, and stored under appropriate conditions until analysis.

The resulting extract was injected into the Rapid Resolution Liquid Chromatography system (RRLC-DAD 1200, Agilent Technologies, Santa Clara, CA, USA). Separation was achieved on a Zorbax Eclipse Plus C18 column (4.6, 150 mm, 5 μm) using a binary solvent system composed of 0.01% formic acid in water (solvent A) and acetonitrile (solvent B) pumped to 100% A at 0 min; 95% A + 5% B at 5 min; 50% A + 50% B at 20 min; washing and re-balancing of the column at 22 min. The chromatograms were retrieved using OpenLab ChemStation software (version 2.15.26).

Identification of compounds was based on the comparison of retention times and UV-Vis spectra with those of reference standards, and monitored at wavelengths of 280, 320, and 370 nm. Compounds such as caffeic acid, chlorogenic acid, chrysin, *p*-coumaric acid, *m*-coumaric acid, *o*-coumaric acid, ferulic acid, gallic acid, *p*-hydroxybenzoic acid, 3-hydroxybenzoic acid, 2,5-dihydroxybenzoic acid, kaempferol, luteolin, naringin, quercetin, rutin, shikimic acid, syringic acid, and vanillic acid were used for this purpose. Concentrations were expressed in milligrams per 100 g of dry weight (100 g of dry weight). Each analysis was performed in duplicate or triplicate to ensure reproducibility and accuracy.

### 2.4. Antioxidant Activity Analyses

The antioxidant potential of the samples was assessed using the ABTS and DPPH methods, following the protocol described by Coyago-Cruz et al. [21]. The extraction was performed in triplicate. 20 mg of freeze-dried powder was combined with 2 mL of methanol and vortexed, then subjected to sonication in an FS60 ultrasonic bath (Fisher Scientific Inc., Waltham, MA, USA) for 3 min. The solids were separated by centrifugation at 14,000× *g* rpm and 4 °C for 4 min. The liquid was passed through a 0.45 µm PVDF filter, and the resulting filtrate was collected for analysis.

The ABTS•+radical was generated by mixing 2.45 mM potassium persulfate with 7 mM ABTS and leaving the mixture to stand in the dark for 16 h. The resulting solution had an absorbance of 0.70 ± 0.02 at 734 nm. To quantify the sample, 10 μL of the extract or standard was placed in a microplate well containing 200 μL of the ABTS radical. The mixture was shaken for 15 min in the dark. Absorbance was measured at 734 nm using a microplate reader (Agilent Technologies, Santa Clara, CA, USA) in spectrophotometric mode.

To quantify antioxidant activity using the DPPH method, 20 μL of the extract or standard was added to a microplate well containing 280 μL of DPPH radical solution (10 mg of DPPH dissolved in 50 mL of HPLC-grade methanol). The plate was agitated for 30 min in the dark before the absorbance was measured at 515 nm using a spectrophotometer with a microplate reader.

Trolox was employed as the calibration standard in both assays diluted within the range of 0.2 to 0.7 mM. The results were expressed as millimoles of Trolox equivalents per 100 g of dry weight (mmol TE/100 g DW).

### 2.5. Antimicrobial Activity Analyses

The extract, which had a high phenol content, was prepared by mixing 2.5 g of lyophilised powder (Separate the pulp, peel, and seed) with 25 mL of 50% ethanol. The mixture was homogenised and stirred using ultrasound for six minutes. The supernatant was recovered by centrifugation at 7500× *g* rpm for 5 min at 4 °C. The residue was then re-extracted twice more with the same amount of solvent. The extract was rotary evaporated at temperatures not exceeding 40 °C to remove the ethanol, and the residue was frozen and freeze-dried. The freeze-dried extract was then recovered and stored frozen until analysis [25].

#### 2.5.1. Antibacterial Activity

The bacterial inoculum was prepared in brain–heart infusion broth (BHI) to a final cell density of 5 × 10^5^ CFU/mL. The microbial density was adjusted spectrophotometrically. Additionally, Streptomycin sulphate salt (1400 µg/mL) was used as a control for growth inhibition at the recommended working concentrations for the tested strains. Both BHI alone and supplemented with the extracts at different concentrations were used as blanks.

Extract sensitivities was evaluated using the broth microdilution method according to the Clinical and Laboratory Standards Institute (CLSI) guidelines, with minor modifications [26,27]. Lyophilised extracts (200–400 mg) were reconstituted in 2 mL of sterile water to obtain the initial concentration. Then, 100 µL of each extract solution was mixed with 100 µL of Brain Heart Infusion (BHI) medium, and serial two-fold dilutions were subsequently performed directly in the microplate. A volume of 20 µL of bacterial suspension was added to each well, resulting in a final inoculum density of 1.5 × 10^8^ CFU/mL and a total well volume of 220 µL. The plates were incubated at 37 °C for 24 h. The minimum inhibitory concentration (MIC) was determined by adding 20 μL of 2,3,5-triphenyltetrazolium chloride (TTC) and incubating at 37 °C for 2 h. The MIC was defined as the lowest final concentration of the extract in the wells that completely inhibited visible microbial growth. All assays were performed in at least triplicate.

#### 2.5.2. Antifungal Activity

The fungal inoculum was prepared in YPD to a final cell density of 5 × 10^2^ CFU/mL. The microbial density was adjusted spectrophotometrically. Additionally, Fluconazole (1500 μg/mL) was used as a control for growth inhibition at the recommended working concentrations for the tested strains. Both YPD alone and supplemented with the extracts at different concentrations were used as blanks.

Extract sensitivities were assessed using the broth microdilution method according to the Clinical and Laboratory Standards Institute (CLSI) guidelines, with minor modifications [26,27,28]. Lyophilised extracts (200–400 μg) were reconstituted in 2 mL of sterile water to obtain the initial concentration. Then, 100 µL of each extract solution was mixed with 100 µL of Yeast extract Peptone Dextrose brith, and serial two-fold dilutions were subsequently performed directly in the microplate. Later, 20 µL of the yeast suspension was added to each well, resulting in a final inoculum density of 5 × 10^5^ CFU/mL, with a total well volume of 220 µL. The plates were incubated at 37 °C for 24 h. The minimum inhibitory concentration (MIC) was determined by adding 20 μL of 2,3,5-triphenyltetrazolium chloride (TTC) and incubating at 37 °C for 2 h. The MIC was defined as the lowest final concentration of the extract in the wells that completely inhibited visible microbial growth.

After 24 h of incubation, 4 µL from each well were collected and inoculated onto a Petri dish containing Sabouraud dextrose agar (SDA), which had been previously marked with a grid on its underside. Each aliquot was placed in a designated section of the grid to ensure sample traceability. The dish was then incubated at 37 °C for an additional 24 h to confirm yeast growth inhibition by the tested extracts. The gridded layout enabled direct visual comparison, highlighting differences in colony density, pigmentation, and morphology. This qualitative assessment provided complementary insights to the quantitative data obtained. All assays were performed in at least triplicate.

### 2.6. Anticancer Activity

HeLa (human cervical carcinoma, ATCC No. CCL-2, RRID: CVCL_0030), HCT116 (human colorectal carcinoma (human breast adenocarcinoma, ATCC No. CCL-247, CVCL_0291), HepG2 (human hepatoma, ATCC No. HB-8065, RRID: CVCL_0027), and NIH3T3 (mouse NIH/Swiss embryo fibroblasts, ATCC No. CRL-1658, RRID: CVCL_0594) cell lines were obtained from the American Type Culture Collection (ATCC, Manassas, VA, USA). THJ29T (human thyroid carcinoma, Cat. No. T8254, RRID: CVCL_W922) was obtained from Applied Biological Materials Inc. (ABM, Richmond, BC, Canada). All cell lines were cultured in Dulbecco’s Modified Eagle’s Medium/Nutrient Mixture F-12 Ham (DMEM/F12) (Corning, Corning in Manassas, VA, USA) supplemented with 10% fetal bovine serum (FBS) (Eurobio, Les Ulis, France) and 1% penicillin/streptomycin (Thermo Fisher Scientific, Gibco, Miami, FL, USA) and maintained at 37 °C in a humidified atmosphere with 5% CO_2_.

To evaluate the impact of chicle extracts—obtained separately from the pulp, peel, and seed at M100%—on cellular proliferation, cells were seeded in 96-well plates at a density of 1 × 10^4^ cells per well, based on the growth characteristics of the cell lines employed and ensuring sufficient proliferation during the assay timeframe while avoiding premature confluence. After adherence, they were treated for 72 h with 100 μL of each extract at concentrations ranging from 0.04 to 5 mg/mL. Extracts were dissolved in 100% DMSO, ensuring that the final DMSO concentration in the wells remained below cytotoxic levels to avoid interference with cell viability. Following the incubation period, cell viability was assessed using the MTT assay following standard protocols. Briefly, 10 μL of MTT solution (5 mg/mL) was added to each well, and the plates were incubated for 1–2 h at 37 °C in a humidified atmosphere. Subsequently, the supernatant was carefully aspirated, and 50 μL of DMSO was added to each well to dissolve the resulting formazan crystals. The plates were gently agitated for 2 min to ensure complete solubilization, after which the absorbance was measured at 570 nm using the Cytation 5 multi-mode detection system (BioTek, Winooski, VT, USA). Each concentration was tested in quadruplicate across a minimum of four independent experiments, with results expressed as mean ± standard deviation. To determine the concentration required to inhibit 50% of cell proliferation (IC_50_), dose–response curves were generated using GraphPad Prism 10.2 (GraphPad Software, Inc.). The therapeutic index (TI) was calculated by dividing the IC_50_ value obtained for non-tumour cells (NIH3T3) by that of each tumour cell line.

### 2.7. Haemolytic Activity

The haemolytic activity of the *L. edulis* fruit from the pulp, peel, and seed at M100% was assessed following a previously established protocol. Briefly, ten millilitres of defibrinated sheep blood were subjected to three consecutive washes with PBS 1×. Following these washes, a 1% erythrocyte suspension in PBS 1× was prepared. This erythrocyte suspension was subsequently mixed in a 1:1 ratio with the different extract concentrations, positive controls (10% Triton X-100), or negative controls (DMSO 2.5%) in a 96-well polypropylene plate. The mixture was incubated at 37 °C for 1 h. Post incubation, the samples were centrifuged for 5 min at 1700× *g*. The supernatant was then carefully transferred to a transparent, flat-bottom 96-well plate for absorbance measurement at 405 nm using a Cytation 5 multi-mode plate reader (BioTek). Each experiment included three technical replicates, and the entire procedure was repeated three times. For each sample, the haemolysis rate was calculated according to the formula:

$$HR\%=\frac{ODtest-ODneg}{ODpos-ODneg}*100$$

### 2.8. Statistical Analysis

Data analysis was conducted using Statgraphics Centurion XVII, RStudio (version 4.3.3) and SigmaPlot (version 14.0). Results are reported as mean values accompanied by the standard deviation. A one-way analysis of variance (ANOVA) was applied to assess differences among group means, with post hoc comparisons performed using Tukey’s test at a significance level of *p* < 0.01. Pearson correlations were also calculated at a 99% confidence level to examine relationships across various stages of physiological development.

To determine which variables had the most significant influence on the maturity stage, a principal component analysis (PCA) was performed. This multivariable approach encompassed all measured parameters, including mineral content, ascorbic acid, carotenoid content, phenolics, organic acids, antioxidant capacity (assessed by the DPPH and ABTS methods), and antimicrobial activity. Since the variables differed in units and magnitude, the data were standardised by centring on the mean and scaling to unit variance, resulting in a mean of 0 and a variance of 1 for each variable. This normalisation ensured equal contribution of all parameters to the multivariance model and prevented bias due to differences in measurement scales.

## 3. Results

### 3.1. Physico-Chemical Characteristics

Table 1 shows the average physicochemical and mineral composition values of the chicle fruit at four stages of ripeness, evaluated in different parts of the fruit (pulp, peel, and seed). The pH value ranged from 2.4 in the pulp at M80% and M100% to 6.0 in the peel (M0%) and the seed (M50%). Soluble solids values ranged from 0.1 °Brix in seeds at M80% to 18.6 °Brix in pulp at M100%. Total titratable acidity ranged from 0.1% in seeds at M80% and M100%, and in the peel at M0%, to 2.8% in the pulp at M80%.

Moisture content ranged from 58.1% in seeds at M100% to 81.3% in pulp at M50%, while ash content varied from 0.5% in the peel at M0% to 1.4% in the peel at M50%. Regarding minerals, calcium content varied from 51.5 mg/100 g DW in pulp at M80% to 404.4 mg/100 g DW in the peel at M0%. Iron values ranged from undetectable levels to 12.9 mg/100 g DW in M0% in the pulp. Potassium values ranged from 103.1 mg/100 g DW in seeds at M50% to 824.9 mg/100 g DW in the peel at M100%. Finally, sodium concentrations ranged from 0.6 mg/100 g DW in seeds at M50% to 59.5 mg/100 g DW in pulp at M0%.

### 3.2. Analysis of Bioactive Compounds and Antioxidant Activity

Table 2 shows the results of the analysis of the bioactive compounds in chicle fruit, including vitamin C, organic acids, carotenoids, and phenolic compounds. The table shows the concentrations of each compound individually and the total concentration of each metabolite group. It also demonstrates the antioxidant activity, as determined using the ABTS and DPPH methods.

Vitamin C concentrations ranged from 0.3 mg/100 g DW in M100% seeds to 3.0 mg/100 g DW in M100% pulp. Total organic acid concentrations ranged from 304.9 mg/100 g DW in the seeds of the M50% sample to 3869.1 mg/100 g DW in the peel of the M0% sample. Citric acid concentrations ranged from 85.0 mg/100 g DW in the peel at M0% to 1873.4 mg/100 g DW in the pulp at M80%. Malic acid values ranged from 10.0 mg/100 g DW in the peel at M100% to 3090.8 mg/100 g DW in the pulp at M0%. Tartaric acid ranged from 13.7 mg/100 g DW in seeds at M50% to 587.0 mg/100 g DW in pulp at M0%.

Regarding carotenoids, α-carotene, β-carotene, lutein and zeinoxanthin were mainly identified. The total carotenoid content, calculated as the sum of the individual compounds, ranged from 0.1 mg/100 g DW in the pulp at M0% to 44.4 mg/100 g DW in the peel at M50%. A-Carotene was found to range from 0.4 mg/100 g DW in the seeds at M100% to 14.6 mg/100 g DW in the pulp at M100%. Β-Carotene was found to range from 0.5 mg/100 g DW in the seeds at M100% to 35.0 mg/100 g DW in the peel at M50%. Lutein concentrations ranged from 0.1 mg/100 g DW in seeds at M100% and pulp at M0% to 3.4 mg/100 g DW in seeds at M0%. Finally, zeinoxanthin values ranged from 0.1 mg/100 g DW (in pulp at M50%, peel at M0% and seeds at M0%) to 0.2 mg/100 g DW (in pulp at M80% and M100%).

The majority of the phenolic compounds detected were gallic acid, syringic acid, chlorogenic acid, naringenin, kaempferol, quercetin glycoside and quercetin. The total phenol content, calculated as the sum of the individual compounds, ranged from 132.5 mg/100 g DW in the seeds at M80% to 11,178.1 mg/100 g DW in the peel at M100%. Gallic acid ranged from 2.7 mg/100 g DW in the peel at M50% to 50.6 mg/100 g DW in the pulp at M100%. Syringic acid ranged from 6.0 mg/100 g DW in the pulp at M0% to 26.6 mg/100 g DW in the seeds at M0%. Chlorogenic acid ranged from 7.4 mg/100 g DW in the peel at M0% to 333.2 mg/100 g DW in the peel at M50%. Naringenin showed a wide range, from 535.0 mg/100 g DW in the pulp at M100% to 10,890.9 mg/100 g DW in the peel at M100%. Kaempferol values ranged from 5.4 mg/100 g DW in seeds at M50% to 40.9 mg/100 g DW in seeds at M0%. Quercetin glycoside varied from 21.9 mg/100 g DW in pulp at M0% to 50.3 mg/100 g DW in seeds at M0%, while quercetin ranged from 53.6 mg/100 g DW in seeds at M80% to 758.4 mg/100 g DW in the peel at M0%.

Finally, antioxidant activity showed significant variation depending on the method used. In the ABTS assay, values fluctuated between 3.1 and 6.4 mmol ET/100 g DW in seeds at M100% and M0%, respectively. By contrast, in the DPPH assay, concentrations ranged from 2.2 mmol ET/100 g DW in seeds at M0% to 63.3 mmol ET/100 g DW in the peel at M50%.

### 3.3. Antimicrobial Activity Analyses

The antimicrobial activity of extracts obtained from pulp, peel, and seeds of chicle fruits at four ripening stages (M0% to M100%) was assessed against reference bacterial strains, such as *Escherichia coli* ATCC 8739, *Pseudomonas aeruginosa* ATCC 9027, *Staphylococcus aureus* ATCC 6538P, and *Streptococcus mutans* ATCC 25175 and fungi against *Candida albicans* ATCC 1031 and *Candida tropicalis* ATCC 13803. The minimal inhibitory concentration (MIC, mg/mL) values are summarised in Table 3.

### 3.4. Anticancer Activity

The antiproliferative activity of chicle fruit extracts—derived from the pulp, peel, and seeds at M100% maturity—was evaluated using the MTT assay across a panel of tumour cell lines (HeLa, HCT116, THJ29T, and HepG2) and non-tumour fibroblasts (NIH3T3) (Table 4).

The extract derived from the seeds did not exhibit activity at the tested concentrations. In contrast, both pulp and peel extracts demonstrated measurable antiproliferative effects, with IC_50_ values ranging from 1.34 to 5.24 mg/mL. The peel extract showed stronger activity overall, particularly against cervical carcinoma and hepatoma cells (HeLa and HepG2). The pulp extract showed lower potency, with the highest activity observed in colorectal carcinoma cells (HCT116). Thyroid carcinoma cells (THJ29T) are the least responsive overall, as they exhibit high IC_50_ values for both extracts, indicating lower sensitivity across the board compared to other cell lines.

Therapeutic index (TI) values, calculated by comparing IC_50_ values in NIH3T3 non-tumour cells to those in tumour cells, were generally low for the pulp extract (0.4–0.8), suggesting limited selectivity. In contrast, the peel extract displayed improved TI values, reaching 2.1 in hepatoma cells and 1.9 in cervical carcinoma cells, indicating a more favourable therapeutic profile. Nevertheless, the overall narrow TI values suggest that while some selectivity is present, especially in the peel extract, the therapeutic window remains limited.

### 3.5. Hemolytic Activity

The hemolytic potential of *Lacmellea edulis* fruit extracts was assessed in vitro using defibrinated sheep erythrocytes exposed to five serial concentrations (2500, 1250, 625, 312.5, and 156.25 µg/mL) of each fruit fraction: peel, pulp, and seed extracts at M100% maturity. Erythrocyte membrane integrity was evaluated spectrophotometrically by measuring the release of haemoglobin at 405 nm after a 1 h incubation at 37 °C.

**Figure 2:**
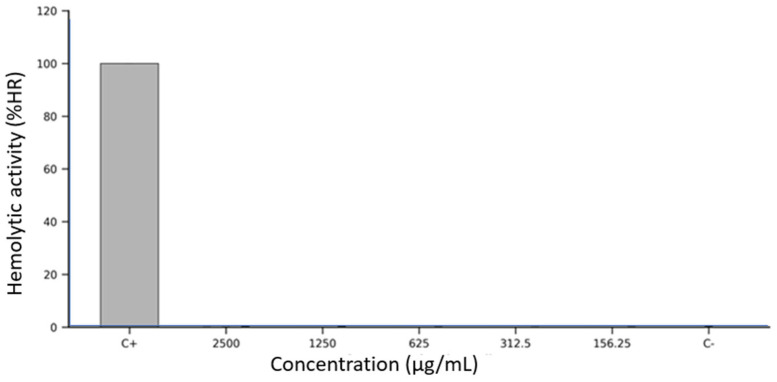
Hemolytic activity (%HR) of *Lacmellea edulis* fruit extracts on sheep erythrocytes at different concentrations (2500–156.25 µg/mL), compared to positive (C+: 10% Triton X-100) and negative (C−: 2.5% DMSO) controls. All extract-treated samples exhibited hemolysis levels below 1%, comparable to those of the negative control, indicating no membrane-disruptive effect under the tested conditions.

As shown in Figure 2, all three extracts demonstrated consistently low levels of hemolysis across the entire concentration range tested. The peel extract produced hemolysis values below 1% at all concentrations, closely resembling the negative control (DMSO 2.5%), with no apparent dose-dependent effect. The seed extract followed an identical pattern, with hemolysis percentages remaining below 1% and minimal variability across replicates. The pulp extract also exhibited minimal hemolytic activity. In every condition tested, the measured absorbance values corresponded to less than 1% hemolysis, confirming the non-lytic nature of the extract on erythrocyte membranes. Error bars in Figure 2 indicate low intra- and inter-assay variability, highlighting the assay’s reproducibility and robustness. In contrast, the positive control (10% Triton X-100) induced erythrocyte lysis, validating the assay system’s sensitivity.

Overall, none of the *L. edulis* extracts produced significant hemolysis under the assay conditions, even at the highest tested concentration (2500 µg/mL), and no fraction exhibited a concentration-dependent increase in erythrocyte disruption.

### 3.6. Statistical Analysis

**Figure 3:**
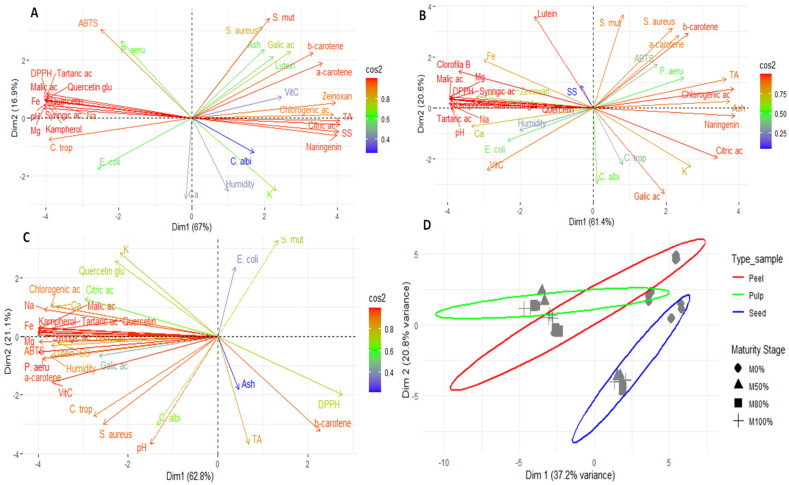
Exploratory multivariate analysis using PCA; (**A**) pulp; (**B**), peel; (**C**) seed and (**D**) Global analysis. Note: SS, soluble solid; TA, titratable total acidity; Ca, calcium; Fe, Iron; K, potassium; Mg, magnesium; Na, sodium; VitC, vitamin C; ac, acid; Zeinoxan, zeinoxanthin; Quercetin glu, Quercetin glucoside; E. coli, *Escherichia coli*; P. aeru, *Pseudomonas aeruginosa*; S. aureus, *Staphylococcus aureus*; S. mut, *Staphylococcus mutans*; C. albi, *Candida albicans*; C. trop, *Candida tropicalis*.

Figure 3 shows the results of the principal component analysis (PCA) of the pulp, peel, seeds, and global analysis of the fruit. This analysis revealed notable differences in the composition of the fruit’s various parts by identifying patterns of grouping and separation. The results demonstrate that each part of the fruit (pulp, peel, and seeds) has a distinct chemical and functional profile, confirming the internal heterogeneity of *Lacmellea edulis*.

## 4. Discussion

### 4.1. Physico-Chemical

Significant differences were observed in most cases, both between different parts of the fruit and between the same tissues at different stages of ripeness. The results showed that the pH of the pulp, peel and seeds decreased as ripening progressed, while the pulp’s soluble solids increased progressively. Indeed, the pulp of the chicle tree reached concentrations of soluble solids comparable to those of grapes used for winemaking (19–25 °Brix) and close to the values reported in table grapes (13–14 °Brix) [29,30]. However, the soluble solids reported in this study (18.6 °Brix in M100% pulp) were lower than those in *Lacmellea standleyi* (25.1 °Brix at maturity) [20] and similarly another study in *Lacmellea edulis* (17.1 °Brix at maturity) [17].

Total titratable acidity was also high in the more mature stages, at 2.7–2.8%, characterising chicle fruit as acidic. These changes are consistent with the biochemical processes of ripening, which involve coordinated transformations in carbohydrate and organic acid metabolism, as described in *Prunus* species, where acidity decreases as the fruit progresses from the immature to the ripe stage [31]. The total titratable acidity of the ripe fruit examined in this study was lower than the values reported by other authors for *L. edulis* (3%) [17] and higher than the value reported for unripe fruit in *Lacmellea oblongata* (0.1%) [21].

No definite pattern of change in moisture content was identified between the different parts, except in the seeds, where a progressive decrease was observed as they matured. This behaviour is associated with natural physiological processes that promote the longevity and stability of seeds in storage. Indeed, moisture sorption isotherms demonstrate the equilibrium between water content and relative humidity, illustrating the impact of water loss during ripening on both physiology and storage potential [31]. This trend is consistent with observations in other fruits, such as *Mespilus germanica*, where moisture content decreases progressively over time during ripening [32].

Regarding ash, the results showed a progressive increase in the pulp of ripe fruit. However, the recorded values were lower than those previously reported for ‘Lechemiel’ (3.8% in the green state, 1.8% in the intermediate state, and 4.5% in the ripe state) [20]. Regarding minerals, the highest concentrations were found in the seeds at M0%, a trend that was also observed in some cases in the pulp and peel. Generally, the relative distribution coincided with that reported in the literature, with potassium being the predominant mineral, followed by calcium and magnesium [20], as reported in unripe *L. oblongata* [21].

### 4.2. Bioactive Compounds and Antioxidant Activity

Vitamin C (ascorbic acid) is an essential antioxidant that is found in high concentrations in plant fruits, which are the main source of vitamin C in the human diet [33]. Thus, when the different parts of the fruit were compared, it was found that the seeds had the lowest vitamin C concentrations, ranging from 0.3 mg/100 g DW in M100% to 1.7 mg/100 g DW in M0%. Furthermore, the concentration of vitamin C in the seeds decreased progressively as the fruit ripened. By contrast, other studies on *Lacmellea standleyi* reported even lower vitamin C values (0.18 mg/100 g DW in the green state, 0.02 mg/100 g DW in the intermediate state and 0.03 mg/100 g DW in the ripe state) [20]. Furthermore, the vitamin C content of the pulp in this study was higher than the non-detectable limit reported for unripe *L. oblongata* [21]. In contrast, a high concentration was reported for *L. lactenscens* in ripe fruit (8.1 mg/100 g DW) [17]. These differences may be due to the regulation of ascorbic acid accumulation in fruit by various factors, including transcription factors, protein interactions, phytohormones, and environmental conditions [33].

The highest concentration of total organic acids was found in the pulp, with a predominance of malic and tartaric acid at the M0% maturity stage. Concentration of citric acid increased in the peel as ripening progressed, while malic acid tended to decrease in this tissue. These compounds play a key role in the characteristic flavour of the pulp and peel of stone fruits [34]. The decrease observed in some metabolites can be attributed to dilution rather than catabolism, given that large amounts of sugars accumulate during ripening, which leads to a reduction in organic acids [35]. Consequently, the higher concentration recorded in the pulp suggests that these compounds are synthesised primarily in this tissue, or imported in an undissociated form, as other authors have suggested. The content of organic acids varies between species and depends on the tissue and stage of development. Thus, the reduction in malic acid during ripening is explained by a combined effect of dilution and metabolic transformation. Additionally, the metabolism of organic acids is interconnected across different parts of the fruit, with close relationships observed between nitrogen metabolism and the malic and citric acid pathways, as previously described in stone fruits [36].

On the other hand, the total concentration of organic acids obtained in this study was similar to that reported for unripe *L. oblongata* fruits (3887.7 mg/100 g DW), in which citric acid accumulation predominated, followed by malic acid [21]. However, these values were lower than those reported for ripe *L. edulis* fruits (8.9 g/100 g DW), for which a predominance of citric and tartaric acids was observed [17]. It is worth noting that multiple factors, including variety, environmental conditions, and interactions with pathogens, significantly influence the concentration and profile of organic acids in fruits. Water stress is one such factor, as it alters the gene expression associated with the metabolism of key acids, such as malic, tartaric, and citric acids. This, in turn, alters the synthesis and activity of the enzymes responsible for their metabolism [37].

Regarding carotenoids, these natural, lipophilic pigments are found in fruits and vegetables, and act as antioxidants, providing health benefits for humans [38]. Thus, a higher concentration was observed in the peel, followed by the pulp, with α-carotene and β-carotene being the predominant forms of carotene. This effect was also observed in other fruits that accumulated higher concentrations of carotenoids in the peel. This process depends on the presence of functional chromoplasts in the tissues, the expression of biosynthetic genes, and environmental factors such as light and temperature [39]. In the pulp, the concentration of these carotenoids increased with ripeness, in line with the biosynthesis of the pigments responsible for colour during ripening [40]. In contrast, the concentration of α-carotene, β-carotene and lutein decreased progressively in the seeds as they ripened. Conversely, the total carotenoid concentration obtained in this study was lower than that reported for unripe *L. oblongata* fruits (4.8 mg/100 g DW), in which β-carotene accumulation predominated [21]. However, these values were lower than those reported for ripe *L. edulis* fruits (284.1 mg/100 g DW), in which α-carotene predominated [17].

Regarding phenolic compounds, the highest concentration was found in the peel, followed by the pulp, and then the seeds. No definite pattern of change was observed in any of the major phenolic compounds. These results are consistent with those reported for *Solanum betaceum*, in which a preferential accumulation of phenols in the peel has been documented [13]. Furthermore, the total phenol concentration obtained in this study was lower than that reported for unripe *L. oblongata* fruits (2423.0 mg/100 g DW), in which chlorogenic acid and naringenin predominated [21]. However, these values were higher than those reported for ripe *L. edulis* fruits (41.1 mg/g DW), for which quercetin accumulation predominated [17]. These differences may be due to the fact that the phenolic content of a plant is influenced by external factors, such as the variety of the plant, the cultivation techniques employed, and the climate [41].

Antioxidant activity also reflected differences between tissues. When evaluated using the ABTS method, the highest activity was found in the peel, followed by the seeds. In contrast, using the DPPH method, only the peel exhibited higher activity. Other studies have shown that the antioxidant activity of unripe *L. oblongata* fruit is 3.6 mmol TE/100 g DW by the DPPH method and 6.6 mmol TE/100 g DW by the ABTS method [21]. These differences can be attributed to the specificity of the assays used. The ABTS method interacts with both hydrophilic and lipophilic compounds, whereas the DPPH method responds mainly to water-soluble molecules, making it more sensitive to phenolic compounds. In particular, the DPPH assay is highly sensitive to molecules with multiple hydroxyl groups, such as phenolic acids, where the number of OH groups significantly influences antioxidant capacity. Conversely, the ABTS assay can react with a broader spectrum of antioxidants, including those that are not recognised by the DPPH assay, such as certain flavanones and dihydrochalcones [42].

### 4.3. Antimicrobial Activity

In *Streptococcus mutans*, the lowest MIC was observed in M1 pulp (2.6 mg/mL), followed by M0 pulp (5.2 mg/mL) and M3 peel/M1 seed (5.3 mg/mL). A similar pattern was identified in *E. coli*, where the lowest MIC was observed in M2 pulp (2.7 mg/mL), followed by M3 peel/M1, M3, and M1 seed (5.3 mg/mL). In pulp, although the phenolic concentration is lower than in peel, the higher load of organic acids (citric) could chelate divalent cations that stabilise the lipopolysaccharide and enhance the entry of phenols [43,44,45], it could also enter the interior of the cells and change the intracellular pH preventing bacterial growth [46], this would explain the lower MIC in pulp surpassing that of peel despite having less phenols. In peel, the high phenolic load (naringenin/quercetin) would favour the disruption of the external barrier and the collapse of gradients [47].

Regarding *P. aeruginosa*, all extracts showed limited antimicrobial effects, with MICs ≥ 21.9 mg/mL. This result is consistent with its poorly permeable outer membrane, efflux pumps, quorum-sensing regulation, and biofilm formation, which limit the effect of phenols and acids to moderate concentrations [48,49]. The small decrease observed in M2 pulp can be attributed to the combination of acids with residual phenols, but it is insufficient to completely overcome *P. aeruginosa’s* barriers.

In the case of *S. aureus*, pronounced differences were observed depending on tissue type and ripening stage. Peel extract at stage M0% exhibited the highest activity (21.3 mg/mL), followed by M100% with similar efficacy. This pattern is consistent with the higher abundance of phenols in peel: naringenin and quercetin are plausible factors responsible for cytoplasmic membrane permeabilisation, enzyme inhibition, and redox alteration, as well as the inhibition of nucleic acid synthesis [50,51,52]. In contrast, seeds were mostly inactive (NE u ≥83.3 mg/mL), consistent with lower phenolic loading/bioavailability.

In the case of *C. albicans*, the MIC values for pulp extracts remained relatively consistent across all ripening stages (21.0–21.9 mg/mL), indicating a modest but uniform antifungal activity. Similarly, peel extracts showed comparable efficacy, with MICs ranging from 21.0 to 21.8 mg/mL. However, seed extracts exhibited significantly lower activity, particularly at M0% and M80% (88.5 and 83.3 mg/mL, respectively). Notably, only M100% seed extract demonstrated moderate antifungal activity (20.8 mg/mL), suggesting a potential accumulation of active constituents at later ripening stages.

Conversely, *C. tropicalis* was more susceptible to the chicle extracts. Pulp extracts showed considerable antifungal activity at M0% (2.6 mg/mL) and M100% (5.3 mg/mL), with a slight decrease in efficacy at intermediate stages. The peel extract at M50% exhibited the most potent inhibition (5.3 mg/mL), while M80% showed reduced activity (43.7 mg/mL). Seed extracts followed a similar trend, with M0%, M50%, and M100% showing moderate activity (MICs between 20.8 and 44.3 mg/mL), whereas M80% was less effective (83.3 mg/mL). This species specificity could reflect differences in wall/membrane composition (ergosterol, mannans) [53] and antioxidant capacity of each fungus, modulating their vulnerability to polyphenols (protein precipitation, metal chelation, redox disruption) such as naringenin and quercetin, which have demonstrated antifungal effect [53,54].

These findings suggest that the antimicrobial potential of chicle fruit extracts is influenced by both the stage of ripeness and the type of tissue. In particular, extracts from the pulp and peel contain bioactive compounds that exhibit promising antibacterial and antifungal activity. Similar results have been reported for other species in the same genus. A study of *L. gracilis* revealed antibacterial activity against *E. coli* and *S. aureus*, as well as antifungal activity against *C. albicans*, in extracts obtained from leaves and branches using various solvents [55]. Consistent with this, research on the unripe fruit of *L. oblongata* reported minimum inhibitory concentrations (MICs) of 31.3 mg/mL for *E. coli*, 83.3 mg/mL for *S. aureus*, 10.4 mg/mL for *S. mutans*, 20.8 mg/mL for *C. albicans*, and 20.8 mg/mL for *C. tropicalis* [21].

### 4.4. Anticancer Activity

To date, no studies have reported the antitumor activity of chicle, making this work the first to explore the antiproliferative properties of its edible fruit. Although other members of the Apocynaceae family have been studied for their antitumoral activity, particularly through extracts or isolated compounds that exhibit selective cytotoxicity against cancer cells, most of this research has focused on non-edible parts such as leaves and roots [56,57,58]. The current findings expand the chemobiological landscape of this family by identifying chicle peel and pulp as sources of moderate cytotoxic activity against tumour cells. While less potent than purified plant-derived compounds, the biological activity observed here is consistent with other non-optimised fruit extracts.

The differences in biological activity of each fruit part align closely with the distinct phytochemical compositions. The peel, which emerged as the most bioactive fraction, is notably rich in phenolic compounds—particularly naringenin, a flavonoid with well-established antitumoral properties. Naringenin has been shown to inhibit cancer cell proliferation, trigger apoptosis via mitochondrial and caspase activation, and disrupt key oncogenic signalling pathways [59,60]. Its abundance in the peel, along with the presence of chlorogenic acid, likely underlies its enhanced activity. In contrast, the seed extract, despite containing quercetin derivatives [61], lacked the broader diversity and concentration of flavonoids and showed no measurable activity. The pulp, though less phenolic-rich than the peel, exhibited the highest organic acid content, notably citric acid, which has been reported to alter cellular metabolism and pH in ways that may inhibit tumour growth [62]. While its antiproliferative effect was less pronounced, this distinct chemical profile suggests a different mode of action compared to the flavonoid-driven cytotoxicity observed in the peel. Notably, only the peel extract demonstrated meaningful tumour selectivity, as reflected by therapeutic index values above 1 in several cancer cell lines.

These findings emphasise the relevance of using not only edible pulp but also fruit by-products such as peels as sources of functional compounds for further pharmacological investigation. The lack of activity in the seeds underscores the importance of targeted phytochemical screening rather than assuming uniformity across fruit tissues. These observations provide a first step towards understanding the antitumoral potential of chicle fruit, positioning the peel as the most promising part for future studies.

### 4.5. Hemolytic Activity

The data presented in Figure 2 reveal a complete absence of hemolytic activity across all fractions of *L. edulis* fruit extracts, even at concentrations as high as 2500 µg/mL. This consistent lack of erythrocyte membrane disruption strongly suggests that the chemical constituents of the peel, pulp, and seed extracts are hemocompatible and do not exhibit the amphipathic or surfactant-like properties typically associated with membrane-permeabilising agents.

Importantly, this hemocompatibility is not only supported by the absence of hemolysis but may also be mechanistically explained by the phytochemical composition of the extracts. As shown in Table 2, all fruit fractions contain elevated levels of phenolic compounds—particularly chlorogenic acid, naringenin, and quercetin—as well as substantial antioxidant activity, as measured by ABTS and DPPH assays. These molecules are widely recognised for their membrane-stabilising properties [63] and are known to protect erythrocytes against oxidative and mechanical stress [64].

Flavonoids such as naringenin and quercetin have been shown to inhibit hemolysis induced by hypochlorous acid and peroxyl radicals, primarily by preserving membrane thiol groups and scavenging reactive oxygen species [65,66]. Furthermore, naringenin can intercalate into lipid bilayers, increasing membrane fluidity and enhancing the mechanical stability of erythrocytes [64,67]. Likewise, chlorogenic acid has been reported to localise at the lipid–water interface of membranes, where it neutralises lipid peroxyl radicals and prevents peroxidative damage [68].

These protective mechanisms are likely relevant in this context. The peel extract, in particular, exhibits remarkably high levels of naringenin (approximately 10 g/100 g DW, Table 2), which could contribute substantially to the observed lack of hemolysis. Additionally, Table 1 shows that the peel and pulp are moderately rich in organic acids and mineral ions—components that may help maintain osmotic stability and ionic balance, further safeguarding erythrocyte integrity. The absence of a dose-dependent increase in hemolysis, as evident in Figure 2, further supports the interpretation that these extracts do not destabilise membranes, even under supraphysiological conditions.

While these in vitro findings provide a compelling indication of hemocompatibility, it is essential to acknowledge the inherent limitations of the erythrocyte model. Red blood cells, lacking nuclei and organelles, represent a simplified system that does not capture the full scope of toxicological responses. Future studies should explore the cytotoxic, genotoxic, and systemic effects of these extracts in nucleated human cells and in vivo systems.

Thus, the results shown in Figure 2, when interpreted in the context of the phytochemical richness outlined in Table 1 and Table 2, suggest that *L. edulis fruit* extracts are not only non-hemolytic but may actively support membrane stability through the action of phenolic antioxidants. These findings provide a strong rationale for further exploration of their therapeutic or nutraceutical potential, with an emphasis on mechanistic toxicology and bioactivity profiling.

### 4.6. Statistical Analysis

Principal component analysis (PCA) of the pulp (Figure 3A) revealed that Dim1 accounted for 67% of the variability, while Dim2 accounted for a further 16.9%. Together, they accounted for 83.9% of the total variability. In the right quadrant, carotenoids (α- and β-carotene, lutein and zeaxanthin) and certain phenolic compounds (chlorogenic acid, gallic acid and naringenin) were found, which are closely associated with high activity against *S. mutans* and *S. aureus*, as well as higher acidity and soluble solids values. This pattern indicates that ripening increases the presence of compounds associated with antimicrobial activity and sensory attributes, as previously proposed by other researchers [69]. The left quadrant contained organic acids (malic and tartaric acids) and compounds such as quercetin glycoside, which are associated with DPPH antioxidant activity, as well as minerals including iron, sodium, and magnesium. The ABTS method was positioned independently on the upper axis, indicating that it captures both hydrophilic and lipophilic antioxidants without closely correlating with a single group of metabolites. Finally, *C. albicans* and moisture were projected towards the lower part and were associated with calcium and potassium. This suggests that the susceptibility of this fungus may be more closely linked to storage factors and water content than to specific metabolites.

In the case of PCA for the peel (Figure 3B), Dim1 explained 61.4% of the variability, while Dim2 explained 20.6%, giving a total of 82%. The behaviour of the variables showed certain similarities to that observed in the pulp, albeit with a more heterogeneous profile. This is consistent with the protective function of the peel and its high phenolic content, as suggested by other authors [13,18].

The PCA for seeds showed that Dim1 explained 62.8% of the total variation, while Dim2 explained 21.1%, for a total of 83.9% (Figure 3C). Most phenolic compounds and organic acids were grouped in the left quadrant, alongside minerals such as sodium, calcium, and magnesium. Vitamin C, moisture and ABTS antioxidant activity were also located in association with inhibition against *P. aeruginosa*, *S. aureus* and *C. tropicalis*. By contrast, β-carotene, DPPH antioxidant activity, ash and titratable acidity were found on the right, indicating a shift towards lipophilic compounds in the antioxidant profile in more advanced stages of ripeness.

Finally, the overall PCA, grouping by tissue type (peel, pulp, and seeds) and maturity status (M0–M100%), explained 58% of the total variation (Dim1 = 37.2%; Dim2 = 20.8%; Figure 3D). The results showed that the peel formed a well-defined group with a differentiated and heterogeneous chemical profile, probably influenced by its protective function and high phenolic content. The pulp formed a more central group closer to the median axis with less dispersion, reflecting a more homogeneous chemical profile throughout ripening. The seeds were clearly separated, differing in Dim2, indicating a distinct chemical profile associated with reserve metabolites and defence compounds. Depending on the ripening stage, M0% and M50% tended to cluster in the central region of the peel, while M80% and M100% were more dispersed, suggesting increased chemical variability in the later stages of ripening. In the pulp, the phases remained relatively close, indicating stability in their composition during development. In the seeds, a progressive shift in Dim1 and Dim2 was observed as ripening progressed, reflecting significant changes in composition in terms of phenols, minerals, and antioxidants.

## 5. Conclusions

This study provides the first comprehensive characterisation of the bioactive compounds and associated biological activities in chicle fruit (*Lacmellea edulis*). These were evaluated in different tissues (pulp, peel, and seeds) and stages of ripeness (M0%, M50%, M80%, and M100%). The results showed that high ascorbic acid levels were found in the ripe pulp, organic acids in the immature pulp, carotenoids in the peel, and total phenols in the ripe peel, accompanied by strong antioxidant activity in the M50% peel. Antimicrobial assays showed that pulp was most effective against *E. coli* and *S. mutans*, while peel displayed higher activity against *S. aureus* and *C. tropicalis*. Seeds were generally less effective, except against *C. albicans* at full ripeness. Pulp and peel extracts at M100%, however, showed antiproliferative activity against different tumour lines. The peel was particularly effective against cervical cancer (HeLa) and hepatoma (HepG2) cells, demonstrating greater efficacy and more favourable therapeutic indices. Furthermore, none of the extracts (pulp, peel, and seeds) exhibited significant haemolytic effects on sheep erythrocytes at any concentration, confirming their safety for cell membranes. Multivariate analysis revealed that tissue type is a more decisive differentiating factor than degree of maturity. The pulp showed a homogeneous profile; the peel, a heterogeneous protective profile; and the seeds, a particular profile associated with reserve and defence metabolites. Taken together, these findings highlight *L. edulis* as a promising source of bioactive metabolites with antioxidant, antimicrobial, and antiproliferative potential, supporting its valorisation for functional foods, nutraceuticals, and pharmaceutical applications, while contributing to the sustainable exploitation of Mesoamerican biodiversity.

## Figures and Tables

**Table 1:** Average values of the physicochemical characteristics of chicle at different stages of maturity.

	Pulp				Peel				Seed							
Maturity	M0%	M50%	M80%	M100%	M0%	M50%	M80%	M100%	M0%	M50%	M80%	M100%	A_M0_	A_M50_	A_M80_	A_M100_
**Weight (g)**	14.6 ± 3.5 ^a^	16.9 ± 4.8 ^a^	15.4 ± 2.2 ^a^	12.5 ± 0.7 ^b^	8.7 ± 2.1 ^a^	2.8 + 0.9 ^c^	2.9 + 0.7 ^c^	3.4 + 0.6 ^b^	5.8 ± 1.4 ^a^	0.7 + 0.1 ^c^	0.6 + 0.1 ^c^	1.1 + 0.6 ^b^	**	ns	***	***
**pH**	5.8 ± 0.1 ^a^	2.6 ± 0.1 ^b^	2.4 ± 0.1 ^c^	2.4 ± 0.0 ^c^	6.0 ± 0.0 ^a^	3.0 ± 0.0 ^c^	3.0 ± 0.0 ^c^	3.5 ± 0.0 ^b^	5.8 ± 0.1 ^ab^	6.0 ± 0.0 ^a^	5.5 ± 0.0 ^b^	5.0 ± 0.0 ^b^	ns	***	***	***
**SS (°Brix)**	2.0 ± 0.0 ^c^	16.9 ± 1.0 ^b^	17.5 ± 1.0 ^ab^	18.6 ± 0.4 ^a^	2.0 ± 0.0 ^a^	2.0 ± 0.0 ^a^	2.1 ± 0.1 ^a^	1.7 ± 0.3 ^b^	2.0 ± 0.0 ^a^	1.0 ± 0.0 ^b^	0.1 ± 0.0 ^c^	1.0 ± 0.1 ^b^	ns	***	***	***
**TA (%)**	0.2 ± 0.0 ^c^	2.3 ± 0.1 ^b^	2.8 ± 0.1 ^a^	2.7 ± 0.1 ^a^	0.1 ± 0.0 ^d^	2.1 ± 0.1 ^a^	1.7 ± 0.1 ^b^	1.2 ± 0.1 ^c^	0.2 ± 0.0 ^b^	0.6 ± 0.0 ^a^	0.1 ± 0.0 ^c^	0.1 ± 0.0 ^c^	ns	***	***	***
**Humidity (%)**	75.9 ± 3.8 ^b^	81.3 ± 2.5 ^a^	75.7 ± 1.1 ^b^	77.2 ± 0.2 ^ab^	75.2 ± 0.5 ^a^	66.2 ± 6.5 ^b^	68.6 ± 2.5 ^b^	75.6 ± 0.2 ^a^	75.9 ± 3.8 ^a^	61.5 ± 1.1 ^b^	54.7 ± 4.9 ^d^	58.1 ± 0.6 ^c^	ns	**	***	***
**Ash (%)**	0.7 ± 0.0 ^b^	0.7 ± 0.1 ^b^	0.8 ± 0.2 ^b^	1.1 ± 0.3 ^a^	0.5 ± 0.1 ^c^	1.4 ± 0.5 ^a^	1.1 ± 0.1 ^b^	0.9 ± 0.1 ^b^	0.7 ± 0.0 ^ab^	0.8 ± 0.1 ^a^	0.9 ± 0.1 ^a^	0.6 ± 0.4 ^b^	*	ns	***	ns
**Mineral (mg/100 g DW)**																
**Ca**	151.0 ± 34.2 ^b^	339.3 ± 9.2 ^a^	51.5 ± 8.9 ^c^	122.6 ± 14.4 ^b^	404.4 ± 4.2 ^a^	129.3 ± 1.9 ^c^	113.4 ± 3.6 ^d^	281.4 ± 35.7 ^b^	185.4 ± 80.5 ^a^	52.9 ± 0.2 ^d^	57.9 ± 12.2 ^c^	84.5 ± 5.8 ^b^	***	*	*	**
**Fe**	12.9 ± 0.5	lnd	lnd	lnd	5.3 ± 0.9 ^a^	3.0 ± 0.1 ^b^	2.5 ± 0.2 ^c^	0.1 ± 0.0 ^d^	10.9 ± 2.3	lnd	lnd	lnd	**			
**K**	535.2 ± 38.0 ^d^	713.2 ± 12.8 ^a^	635.3 ± 14.6 ^b^	609.5 ± 8.0 ^c^	449.5 ± 42.1 ^d^	568.4 ± 18.1 ^c^	661.1 ± 19.0 ^b^	824.9 ± 97.0 ^a^	411.8 ± 20.7 ^a^	103.1 ± 0.4 ^d^	284.5 ± 5.0 ^c^	340.6 ± 37.2 ^b^	**	***	***	***
**Mg**	543.7 ± 27.5 ^a^	84.2 ± 18.5 ^b^	37.8 ± 1.7 ^d^	44.8 ± 0.9 ^c^	523.6 ± 6.5 ^a^	60.8 ± 1.0 ^c^	65.9 ± 6.3 ^b^	33.0 ± 6.8 ^d^	752.7 ± 35.8 ^a^	65.9 ± 11.3 ^c^	64.5 ± 2.4 ^c^	74.8 ± 2.1 ^b^	**	ns	***	***
**Na**	59.5 ± 1.0 ^a^	7.4 ± 0.0 ^c^	10.4 ± 1.3 ^b^	7.7 ± 1.6 ^c^	56.6 ± 8.4 ^a^	6.5 ± 0.2 ^d^	8.0 ± 1.5 ^c^	11.5 ± 0.4 ^b^	50.3 ± 1.2 ^a^	0.6 ± 0.0 ^d^	8.1 ± 0.7 ^c^	10.3 ± 2.8 ^b^	*	***	ns	ns

Note: SS, soluble solids; TA, titratable total acidity; lnd, undetectable limit. Different lowercase letters indicate significant differences between stages of ripeness within each part of the fruit under study. Asterisks indicate statistical differences between the various parts of the fruit at the same stage of ripeness: 0% (A_M0_), 50% (A_M50_), 80% (A_M80_) and 100% (A_M100_). ns indicates not significant, while * indicates *p* < 0.1, ** indicates *p* < 0.01 and *** indicates *p* < 0.001.

**Table 2:** Average values of bioactive compounds of chicle at different stages of maturity.

	Pulp				Peel				Seed							
	M0%	M50%	M80%	M100%	M0%	M50%	M80%	M100%	M0%	M50%	M80%	M100%	A_M0_	A_M50_	A_M80_	A_M100_
Vitamin C (mg/100 g DW)	1.7 ± 0.6 ^c^	2.6 ± 0.3 ^b^	1.7 ± 0.0 ^c^	3.0 ± 0.15 ^a^	1.2 ± 0.5 ^a^	0.6 ± 0.0 ^b^	1.1 ± 0.0 ^a^	1.0 ± 0.0 ^a^	1.7 ± 0.5 ^a^	0.9 ± 0.0 ^b^	0.6 ± 0.0 ^c^	0.3 ± 0.0 ^d^	*	***	***	***
Citric acid	269.8 ± 6.8 ^d^	1595.8 ± 32.8 ^c^	1873.4 ± 17.8 ^a^	1667.6 ± 76.4 ^b^	85.0 ± 2.7 ^d^	630.9 ± 27.6 ^c^	988.4 ± 7.7 ^b^	1073.8 ± 12.1 ^a^	309.5 ± 0.0 ^a^	227.3 ± 30.2 ^c^	187.5 ± 6.8 ^d^	282.4 ± 5.1 ^b^	***	***	***	***
Malic acid	3090.8 ± 4.3 ^a^	26.0 ± 1.2 ^c^	18.6 ± 9.6 ^d^	53.6 ± 3.3 ^b^	2912.0 ± 2.3 ^a^	35.8 ± 1.4 ^b^	23.0 ± 4.6 ^c^	10.0 ± 0.5 ^d^	2752.0 ± 0.1 ^a^	63.9 ± 5.1 ^d^	131.9 ± 26.7 ^c^	195.8 ± 26.5 ^b^	**	**	*	**
Tartaric acid	587.0 ± 4.3 ^a^	70.2 ± 0.8 ^c^	94.6 ± 28.8 ^b^	92.4 ± 11.9 ^b^	872.0 ± 2.1 ^a^	30.5 ± 2.3 ^d^	64.6 ± 0.8 ^c^	72.1 ± 7.5 ^b^	451.0 ± 0.1 ^a^	13.7 ± 1.2 ^d^	15.4 ± 0.1 ^c^	22.1 ± 1.3 ^b^	***	***	*	**
Total	3947.6 ± 9.3 ^a^	1689.0 ± 32.4 ^d^	1986.6 ± 20.6 ^b^	1813.5 ± 67.9 ^c^	3869.1 ± 4.9 ^a^	697.2 ± 31.3 ^d^	1076.0 ± 11.5 ^c^	1155.9 ± 12.9 ^b^	3512.5 ± 0.0 ^a^	304.9 ± 36.5 ^c^	334.9 ± 33.3 ^c^	500.3 ± 32.9 ^b^	*	***	***	***
α-Carotene		5.8 ± 0.2 ^c^	8.8 ± 0.9 ^b^	14.6 ± 1.2 ^a^	0.9 ± 0.1 ^d^	6.7 ± 0.7 ^a^	1.2 ± 0.3 ^c^	3.3 ± 0.3 ^b^	11.2 ± 2.7 ^a^	3.3 ± 0.0 ^b^	1.1 ± 0.3 ^c^	0.4 ± 0.0 ^d^	***	**	**	***
β-Carotene		3.4 ± 0.5 ^c^	11.1 ± 1.5 ^b^	14.7 ± 0.1 ^a^	0.8 ± 0.1 ^d^	35.0 ± 0.7 ^a^	8.5 ± 0.7 ^c^	11.3 ± 0.7 ^b^		2.9 ± 0.4 ^a^	1.2 ± 0.1 ^b^	0.5 ± 0.0 ^c^		***	**	***
Lutein	0.1 ± 0.0 ^c^		0.3 ± 0.0 ^a^	0.2 ± 0.0 ^b^	1.4 ± 0.1 ^b^	2.7 ± 0.0 ^a^	0.3 ± 0.0 ^c^	0.3 ± 0.0 ^c^	3.4 ± 0.8 ^a^	0.7 ± 0.0 ^b^	0.3 ± 0.0 ^c^	0.1 ± 0.0 ^d^	***	***	**ns**	**ns**
Zeinoxanthin		0.1 ± 0.0 ^b^	0.2 ± 0.0 ^a^	0.2 ± 0.0 ^a^	0.1 ± 0.0				0.1 ± 0.0							
Total carotenoids	0.1 ± 0.0 ^d^	9.4 ± 0.7 ^c^	20.5 ± 0.6 ^b^	29.8 ± 1.3 ^a^	5.0 ± 0.5 ^d^	44.4 ± 0.0 ^a^	10.1 ± 1.0 ^c^	14.8 ± 0.4 ^b^	15.8 ± 3.1 ^a^	7.0 ± 0.4 ^b^	2.7 ± 0.5 ^c^	1.0 ± 0.0 ^d^	***	***	***	***
Chlorophyll b (mg/100 g DW)					12.5 ± 0.1 ^a^	3.8 ± 0.0 ^b^	0.7 ± 0.1 ^c^									
*Galic acid*	7.6 ± 1.4 ^d^	21.7 ± 2.5 ^b^	16.4 + 1.5 ^c^	50.6 + 5.7 ^a^	2.8 ± 0.3 ^c^	2.7 + 0.1 ^c^	33.5 + 3.5 ^b^	43.2 + 1.9 ^a^	9.9 ± 1.0 ^a^	8.3 + 0.0 ^b^	3.2 + 0.2 ^c^	7.9 + 1.8 ^b^		**	**	**
Syringic acid	6.0 ± 0.2				6.7 ± 0.1				26.6 ± 3.6 ^a^	11.5 ± 1.4 ^b^	10.2 ± 0.7 ^b^	10.9 ± 0.6 ^b^				
Chlorogenic acid	26.9 ± 0.3 ^c^	155.0 ± 36.5 ^ab^	151.0 ± 4.9 ^b^	178.4 ± 33.8 ^a^	7.4 ± 0.8 ^c^	333.2 ± 14.7 ^a^	259.3 ± 25.4 ^ab^	244.0 ± 15.0 ^b^	66.8 ± 9.5 ^a^	14.3 ± 1.6 ^d^	24.4 ± 0.6 ^c^	28.7 ± 2.5 ^b^		**	**	**
Naringenin		544.3 ± 57.4 ^a^	538.4 ± 5.3 ^a^	535.0 ± 82.8 ^a^		9945.7 ± 69.7 ^b^	8749.6 ± 61.8 ^c^	10,890.9 ± 26.8 ^a^						^**^	^**^	^***^
Kampherol	36.1 ± 1.3				36.3 ± 2.2				40.9 ± 1.3 ^a^	5.4 + 0.5 ^c^	5.5 + 0.2 ^c^	7.8 + 0.6 ^b^				
Quercetin glucoside	21.9 ± 1.4				44.6 ± 0.5				50.3 ± 8.7 ^a^	35.1 ± 2.9 ^b^	35.7 ± 3.0 ^b^	50.1 ± 6.6 ^a^				
Quercetin	271.1 ± 12.1				758.4 ± 26.4				455.6 ± 23.2 ^a^	67.3 ± 0.2 ^c^	53.6 ± 0.5 ^d^	79.5 ± 3.5 ^b^				
Total phenolics	369.5 ± 13.7 ^d^	740.7 ± 10.1 ^b^	705.8 ± 1.9 ^c^	764.0 ± 12.2 ^a^	856.2 ± 28.5 ^d^	10,281.6 ± 71.2 ^b^	9042.5 ± 64.6 ^c^	11,178.1 ± 28.5 ^a^	650.1 ± 22.6 ^a^	141.8 ± 6.6 ^c^	132.5 ± 3.8 ^d^	185.0 ± 15.6 ^b^		***	***	***
ABTS	3.3 ± 0.2 ^a^	2.09 ± 0.4 ^b^	2.70 ± 0.4 ^ab^	3.12 ± 0.4 ^a^	3.2 ± 0.0 ^c^	6.3 ± 0.1 ^a^	5.8 ± 0.4 ^ab^	5.3 ± 0.4 ^b^	6.4 ± 0.1 ^a^	4.2 ± 0.8 ^b^	3.4 ± 0.3 ^c^	3.1 ± 0.5 ^c^	**	***	***	***
DPPH	17.1 ± 1.1 ^b^	16.5 ± 1.2 ^b^	18.8 ± 0.7 ^b^	28.8 ± 2.8 ^a^	41.6 ± 1.8 ^c^	63.3 ± 0.7 ^a^	57.0 ± 2.6 ^b^	54.8 ± 1.3 ^b^	2.2 ± 0.1 ^d^	19.1 ± 4.2 ^a^	17.7 ± 0.9 ^b^	15.9 ± 1.4 ^c^	***	***	***	***

Note: Blank spaces in the table indicate that the compound was not present in significant quantities during analysis. Carotenoid and phenol standards that are not reported in the table either showed no significant concentrations or were reported as undetectable. Different lowercase letters indicate significant differences between stages of ripeness within each part of the fruit under study. Asterisks indicate statistical differences between the various parts of the fruit at the same stage of ripeness: 0% (A_M0_), 50% (A_M50_), 80% (A_M80_) and 100% (A_M100_). ns indicates not significant, while * indicates *p* < 0.1, ** indicates *p* < 0.01 and *** indicates *p* < 0.001.

**Table 3:** Minimal inhibitory concentration of chicle at different stages of maturity.

Bacteria or Fungus Strain	Minimal Inhibitory Concentration (mg/mL)											
	Pulp				Peel				Seeds			
	M0%	M50%	M80%	M100%	M0%	M50%	M80%	M100%	M0%	M50%	M80%	M100%
*E. coli* ATCC 8739	10.4	10.6	2.7	5.3	10.7	5.3	10.9	5.3	44.3	5.3	83.3	41.7
*P. aeruginosa* ATCC 9027	83.5	42.3	21.9	84.0	42.6	84.0	43.7	85.0	88.5	84.2	83.3	83.3
*S. aureus* ATCC 6538P	41.8	42.3	43.8	84.0	21.3	84.0	43.7	21.3	84.2	84.2	83.3	83.3
*S. mutans* ATCC 25175	5.2	2.6	10.9	21.0	10.7	21.0	10.9	5.3	11.1	5.3	20.8	83.3
*C. albicans* ATCC 1031	21.0	21.2	21.9	21.0	21.3	21.0	21.8	21.3	88.5	88.5	83.3	20.8
*C. tropicalis* ATCC 13803	2.6	10.6	10.9	5.3	10.7	5.3	43.7	10.7	44.3	42.1	83.3	20.8

**Table 4:** Half maximal inhibitory concentration values (IC_50_) (mg/mL) of chicle against tumour and non-tumour cell lines at 72 h and therapeutic index (TI) values.

	Pulp M100%		Peel M100%		Seeds M100%	
	IC_50_	TI ^a^	IC_50_	TI ^a^	IC_50_	TI ^a^
**HeLa**	5.24 ± 0.70	0.4	1.51 ± 0.35	1.9	NA	--
**HCT116**	2.76 ± 0.30	0.8	2.32 ± 1.14	1.2	NA	--
**THJ29T**	5.05 ± 0.43	0.4	4.38 ± 0.40	0.6	NA	--
**HepG2**	4.62 ± 0.61	0.5	1.34 ± 0.13	2.1	NA	--
**NIH3T3**	2.23 ± 0.30	--	2.81 ± 0.28	--	NA	--

NA: non-active at the tested concentrations. ^a^ IC_50_ = (NIH3T3)/IC_50_ (tumor cell).

## Data Availability

The original contributions presented in this study are included in the article. Further inquiries can be directed to the corresponding author.

## References

[1] Díaz S., Malhi Y. (2022). Biodiversity: Concepts, Patterns, Trends, and Perspectives. Annu. Rev. Environ. Resour..

[2] Durazzo A., Lucarini M., Plutino M., Lucini L., Aromolo R., Martinelli E., Souto E., Santini A., Pignatti G. (2021). Bee Products: A Representation of Biodiversity, Sustainability, and Health. Life.

[3] Baker P., Machado P., Santos T., Sievert K., Backholer K., Hadjikakou M., Russell C., Huse O., Bell C., Scrinis G. (2020). Ultra-Processed Foods and the Nutrition Transition: Global, Regional and National Trends, Food Systems Transformations and Political Economy Drivers. Obes. Rev..

[4] Neira C., Godinho R., Rincón F., Mardones R., Pedroso J. (2021). Consequences of the COVID-19 Syndemic for Nutritional Health: A Systematic Review. Nutrients.

[5] Grajek M., Krupa-Kotara K., Białek-Dratwa A., Sobczyk K., Grot M., Kowalski O., Staśkiewicz W. (2022). Nutrition and Mental Health: A Review of Current Knowledge about the Impact of Diet on Mental Health. Front. Nutr..

[6] Mancuso G., Midiri A., Gerace E., Biondo C. (2021). Bacterial Antibiotic Resistance: The Most Critical Pathogens. Pathogens.

[7] Salam M.A., Al-Amin M.Y., Salam M.T., Pawar J.S., Akhter N., Rabaan A.A., Alqumber M.A.A. (2023). Antimicrobial Resistance: A Growing Serious Threat for Global Public Health. Healthcare.

[8] Coque T., Cantón R., Pérez-Cobas A., Fernández-de-Bobadilla M., Baquero F. (2023). Antimicrobial Resistance in the Global Health Network: Known Unknowns and Challenges for Efficient Responses in the 21st Century. Microorganisms.

[9] Mohammed A., Abdul-Hameed Z., Alotaibi M., Bawakid N., Sobahi T., Abdel-Lateff A., Alarif W. (2021). Chemical Diversity and Bioactivities of Monoterpene Indole Alkaloids (Mias) from Six Apocynaceae Genera. Molecules.

[10] Chelaghma W., Loucif L., Bendahou M., Rolain J. (2021). Vegetables and Fruit as a Reservoir of β-Lactam and Colistin-Resistant Gram-Negative Bacteria: A Review. Microorganisms.

[11] Suriyaprom S., Mosoni P., Leroy S., Kaewkod T., Desvaux M., Tragoolpua Y. (2022). Antioxidants of Fruit Extracts as Antimicrobial Agents Against Pathogenic Bacteria. Antioxidants.

[12] García-Mahecha M., Soto-Valdez H., Carvajal-Millan E., Madera-Santana T., Lomelí-Ramírez M., Colín-Chávez C. (2023). Bioactive Compounds in Extracts from the Agro-Industrial Waste of Mango. Molecules.

[13] Coyago-Cruz E., Guachamin A., Méndez G., Moya M., Martínez A., Viera W., Heredia-Moya J., Beltrán E., Vera E., Villacís M. (2023). Functional and Antioxidant Evaluation of Two Ecotypes of Control and Grafted Tree Tomato (*Solanum betaceum*) at Different Altitudes. Foods.

[14] Kumoro A., Alhanif M., Wardhani D. (2020). A Critical Review on Tropical Fruits Seeds as Prospective Sources of Nutritional and Bioactive Compounds for Functional Foods Development: A Case of Indonesian Exotic Fruits. Int. J. Food Sci..

[15] Lara M., Bonghi C., Famiani F., Vizzotto G., Walker R., Drincovich M. (2020). Stone Fruit as Biofactories of Phytochemicals with Potential Roles in Human Nutrition and Health. Front. Plant Sci..

[16] Dhalaria R., Verma R., Kumar D., Puri S., Tapwal A., Kumar V., Nepovimova E., Kuca K. (2020). Bioactive Compounds of Edible Fruits with Their Anti-Aging Properties: A Comprehensive Review to Prolong Human Life. Antioxidants.

[17] Coyago-Cruz E., Guachamin A., Villacís M., Rivera J., Neto M., Méndez G., Heredia-Moya J., Vera E. (2023). Evaluation of Bioactive Compounds and Antioxidant Activity in 51 Minor Tropical Fruits of Ecuador. Foods.

[18] Coyago-Cruz E., Rodríguez E., Heredia-Moya J., Méndez G. (2024). Functional and Antimicrobial Evaluation of Artocarpus Heterophyllus (Jackfruit) Fruit and Leaves at Different Ripening Stages. Emerging Research in Intelligent Systems.

[19] Nirmal N., Khanashyam A., Mundanat A., Shah K., Babu K., Thorakkattu P., Al-Asmari F., Pandiselvam R. (2023). Valorization of Fruit Waste for Bioactive Compounds and Their Applications in the Food Industry. Foods.

[20] Soto E., Chicué A., Murillo E., Méndez J. (2013). Bioprospecting of Lacmellea Standleyi Fruits (Lechemiel). Rev. Cuba. Plantas Med..

[21] Coyago-Cruz E., Méndez G., Escobar-Quiñonez R., Cerna M., Heredia-Moya J. (2025). Lacmellea Oblongata and Other Undervalued Amazonian Fruits as Functional, Antioxidant, and Antimicrobial Matrices. Antioxidants.

[22] WFO The World Flora Online. https://wfoplantlist.org/plant-list.

[23] (2009). Fruit and Vegetable Products—Determination of Soluble Solids—Refractometric Method.

[24] Berghof-GmbH (2023). Food, Pharma, Cosmetic Microwave Digestion of Spinach.

[25] Coyago-cruz E., Zúñiga-miranda J., Méndez G., Guachamin A., Escobar-Quiñonez R., Barba-Ostria C., Heredia-Moya J. (2025). Relationship between Bioactive Compounds and Biological Activities (Antioxidant, Antimicrobial, Antihaemolytic) of ‘Colcas’ Fruits at Different Stages of Maturity. Antioxidants.

[26] Clinical and Laboratory Standards Institute (CLSI) (2018). M02 Performance Standards for Antimicrobial Disk Suspectibility Tests, Approved Standard-Eleventh Edition. Clinical and Laboratory Standards Institue. Clin. Lab. Stand. Inst..

[27] Balouiri M., Sadiki M., Ibnsouda S. (2016). Methods for In Vitro Evaluating Antimicrobial Activity: A Review. J. Pharm. Anal..

[28] Rex J.H. (2009). CLSI M44-A2 Method for Antifungal Disk Diffusion Susceptibility Testing of Yeasts. Approved Guideline—Second Edition. Clin. Lab. Stand. Inst..

[29] Peters M., Ahlebæk M., Frandsen M., Jørgensen B., Jessen C., Carlsen A., Andersen M., Huang W., Eriksen R. (2025). Investigating the Applicability of a Snapshot Computed Tomography Imaging Spectrometer for the Prediction of °Brix and PH of Grapes. Spectrochim. Acta-Part A Mol. Biomol. Spectrosc..

[30] Joshi V., Reddy S., Rao B. (2022). Physico-Chemical Properties of Juice in Different Wine Varieties of Grape (*Vitis vinifera* L.). Int. J. Plant Soil Sci..

[31] Hay F., Rezaei S., Buitink J. (2022). Seed Moisture Isotherms, Sorption Models, and Longevity. Front. Plant Sci..

[32] Rop O., Sochor J., Jurikova T., Zitka O., Skutkova H., Mlcek J., Salas P., Krska B., Babula P., Adam V. (2011). Effect of Five Different Stages of Ripening on Chemical Compounds in Medlar (*Mespilus germanica* L.). Molecules.

[33] Zheng X., Gong M., Zhang Q., Tan H., Li L., Tang Y., Li Z., Peng M., Deng W. (2022). Metabolism and Regulation of Ascorbic Acid in Fruits. Plants.

[34] Shi Y., Pu D., Zhou X., Zhang Y. (2022). Recent Progress in the Study of Taste Characteristics and the Nutrition and Health Properties of Organic Acids in Foods. Foods.

[35] Batista-Silva W., Nascimento V., Medeiros D., Nunes-Nesi A., Ribeiro D., Zsögön A., Araújo W. (2018). Modifications in Organic Acid Profiles During Fruit Development and Ripening: Correlation or Causation?. Front. Plant Sci..

[36] Famiani F., Bonghi C., Chen Z., Drincovich M., Farinelli D., Lara M., Proietti S., Rosati A., Vizzotto G., Walker R. (2020). Stone Fruits: Growth and Nitrogen and Organic Acid Metabolism in the Fruits and Seeds—A Review. Front. Plant Sci..

[37] Ma W.-F., Li Y.-B., Nai G.-J., Liang G.-P., Ma Z.-H., Chen B.-H., Mao J. (2022). Changes and Response Mechanism of Sugar and Organic Acids in Fruits Under Water Deficit Stress. PeerJ.

[38] González-Peña M., Ortega-Regules A., de Parrodi C., Lozada-Ramirez D. (2023). Chemistry, Occurrence, Properties, Applications, and Encapsulation of Carotenoids—A Review. Plants.

[39] Zhao Y., Yang X., Hu Y., Gu Q., Chen W., Li J., Guo X., Liu Y. (2021). Evaluation of Carotenoids Accumulation and Biosynthesis in Two Genotypes of Pomelo (*Citrus maxima*) During Early Fruit Development. Molecules.

[40] Zhao X., Zhang Y., Long T., Wang S., Yang J. (2022). Regulation Mechanism of Plant Pigments Biosynthesis: Anthocyanins, Carotenoids, and Betalains. Metabolites.

[41] Eseberri I., Trepiana J., Léniz A., Gómez-García I., Carr-Ugarte H., González M., Portillo M. (2022). Variability in the Beneficial Effects of Phenolic Compounds: A Review. Nutrients.

[42] Ranggaini D., Halim J., Tjoe A. (2024). Aktivitas Antioksidan Dengan Metode DPPH Dan ABTS Terhadap Ekstrak Etanol Daun *Amaranthus hybridus* L.. J. Kedokt. Gigiterpadu.

[43] Zhang G., Yang Y., Memon F., Hao K., Xu B., Wang S., Wang Y., Wu E., Chen X., Xiong W. (2021). A Natural Antimicrobial Agent: Analysis of Antibacterial Effect and Mechanism of Compound Phenolic Acid on Escherichia Coli Based on Tandem Mass Tag Proteomics. Front. Microbiol..

[44] Clifton L., Skoda M., Le A., Ciesielski F., Kuzmenko I., Holt S., Lakey J. (2015). Effect of Divalent Cation Removal on the Structure of Gram-Negative Bacterial Outer Membrane Models. Langmuir.

[45] Burel C., Kala A., Purevdorj-Gage L. (2020). Impact of PH on Citric Acid Antimicrobial Activity Against Gram-Negative Bacteria. Lett. Appl. Microbiol..

[46] Silva L., Zimmer K., Macedo A., Trentin D. (2016). Plant Natural Products Targeting Bacterial Virulence Factors. Chem. Rev..

[47] Wang L., Wang M.-S., Zeng X.-A., Xu X.-M., Brennan C. (2017). Membrane and Denomic DNA Dual-Targeting of Citrus Flavonoid Naringenin Against Staphylococcus Aureus. Integr. Biol..

[48] Ude J., Tripathi V., Buyck J., Söderholm S., Cunrath O., Fanous J., Claudi B., Egli A., Schleberger C., Hiller S. (2021). Outer Membrane Permeability: Antimicrobials and Diverse Nutrients Bypass Porins in Pseudomonas Aeruginosa. Proc. Natl. Acad. Sci. USA.

[49] Leveque L., Drockenmuller E., Laktineh I., Alcouffe P., David L., Sudre G., Serghei A. (2025). Nanotubes of Pristine Poly (3-Hexylthiophene) with Modulable Conductive Properties: The Interplay Between Confinement-Induced Orientation and Interfacial Effects. R. Soc. Chem..

[50] Nguyen T.L.A., Bhattacharya D. (2022). Antimicrobial Activity of Quercetin: An Approach to Its Mechanistic Principle. Multidiscip. Digit. Publ. Inst..

[51] Júnior S., Santos J., Campos L., Pereira M., Magalhães N., Cavalcanti I. (2018). Antibacterial and Antibiofilm Activities of Quercetin Against Clinical Isolates of Staphyloccocus Aureus and Staphylococcus Saprophyticus with Resistance Profile. Int. J. Environ. Agric. Biotechnol..

[52] Hou J., Wang H., Pan K., Wu L., Zhao B. (2024). Enhanced Antibacterial Photodynamic Therapy with Qu/Ce6@ZIF-8 Nanoplatform for Staphylococcus Aureus Control in Food Preservation. Food Biosci..

[53] Francisconi R., Ferreira E., Marques M., Fontana A., Lombardi T., Correira M., Palomari D. (2015). Effect of Melaleuca Alternifolia and Its Components on Candida Albicans and Candida Tropicalis. J. US-China Med. Sci..

[54] Duda-Madej A., Stecko J., Sobieraj J., Szymańska N., Kozłowska J. (2022). Naringenin and Its Derivatives—Health-Promoting Phytobiotic Against Resistant Bacteria and Fungi in Humans. Antibiotics.

[55] Basílio A., Simas M., Araújo V., Cristo O., De-Barros G., Pohlit A. (2008). Screening of Amazonian Plants from the Adolpho Ducke Forest Reserve, Manaus, State of Amazonas, Brazil, for Antimicrobial Activity. Mem. Inst. Oswaldo Cruz.

[56] Gonçalves B., Duarte N., Ramalhete C., Barbosa F., Madureira A., Ferreira M. (2025). Monoterpene Indole Alkaloids with Anticancer Activity from Tabernaemontana Species. Phytochem. Rev..

[57] Dhamdhere A., Dhalwade M. (2023). A Detail Review: On Vinca Plant (*Catharanthus roseus*). Int. Res. J. Mod. Eng. Technol. Sci..

[58] Sharma P., Singla N., Kaur R., Bhardwaj U. (2024). A Review on Phytochemical Constituents and Pharmacological Properties of *Catharanthus roseus* (L.) G. Don. J. Med. Plants Stud..

[59] He J., Zhang H. (2023). Research Progress on the Anti-Tumor Effect of Naringin. Front. Pharmacol..

[60] Martínez-Rodríguez O., González-Torres A., Álvarez-Salas L., Hernández-Sánchez H., García-Pérez B., Thompson-Bonilla M., Jaramillo-Flores M. (2020). Effect of Naringenin and Its Combination with Cisplatin in Cell Death, Proliferation and Invasion of Cervical Cancer Spheroids. RSC Adv..

[61] Guo M., Zeng J., Sun Z., Wu X., Hu Z. (2023). Research Progress on Quercetin’s Biological Activity and Structural Modification Based on Its Antitumor Effects. ChemistrySelect.

[62] Icard P., Coquerel A., Wu Z., Gligorov J., Fuks D., Fournel L., Lincet H., Simula L. (2021). Understanding the Central Role of Citrate in the Metabolism of Cancer Cells and Tumors: An Update. Int. J. Mol. Sci..

[63] Oteiza P., Erlejman A., Verstraeten S., Keen C., Fraga C. (2005). Flavonoid-Membrane Interactions: A Protective Role of Flavonoids at the Membrane Surface?. Clin. Dev. Immunol..

[64] Arora A., Byrem T., Nair M., Strasburg G. (2000). Modulation of Liposomal Membrane Fluidity by Flavonoids and Isoflavonoids. Arch. Biochem. Biophys..

[65] Gebicka L., Banasiak E. (2012). Toxicology In Vitro Hypochlorous Acid-Induced Heme Damage of Hemoglobin and Its Inhibition by Flavonoids. Toxicol. Vitr..

[66] Asgary S., Naderi G., Askari N. (2005). Protective Effect of Flavonoids against Red Blood Cell Hemolysis by Free Radicals. Exp. Cardiol..

[67] Tomasz R., Girych M., Bunker A. (2021). Mechanistic Understanding from Molecular Dynamics in Pharmaceutical Research 2: Lipid Membrane in Drug Design. Pharmaceuticals.

[68] Cejas J.P., Rosa A.S., Nazareno M.A., Disalvo E.A., Frias M.A. (2021). Interaction of Chlorogenic Acid with Model Lipid Membranes and Its Influence on Antiradical Activity. BBA-Biomembr..

[69] Coyago-Cruz E., Corell M., Moriana A., Hernanz D., Benítez-González A.M., Stinco C.M., Meléndez-Martínez A.J. (2018). Antioxidants (Carotenoids and Phenolics) Profile of Cherry Tomatoes as Influenced by Deficit Irrigation, Ripening and Cluster. Food Chem..

